# Epithelial-mesenchymal transition-related long noncoding RNAs in gastric carcinoma

**DOI:** 10.3389/fmolb.2022.977280

**Published:** 2022-10-12

**Authors:** Ying-Nan Feng, Bo-Ya Li, Ke Wang, Xiao-Xi Li, Lan Zhang, Xian-Zhe Dong

**Affiliations:** Department of Pharmacy, Xuanwu Hospital, Capital Medical University, Beijing, China

**Keywords:** gastric cancer, epithelial-mesenchymal transition, long non-coding RNAs, signalling pathways, transcription factors, microRNAs

## Abstract

As an evolutionarily phenotypic conversion program, the epithelial-mesenchymal transition (EMT) has been implicated in tumour deterioration and has facilitated the metastatic ability of cancer cells *via* enhancing migration and invasion. Gastric cancer (GC) remains a frequently diagnosed non-skin malignancy globally. Most GC-associated mortality can be attributed to metastasis. Recent studies have shown that EMT-related long non-coding RNAs (lncRNAs) play a critical role in GC progression and GC cell motility. In addition, lncRNAs are associated with EMT-related transcription factors and signalling pathways. In the present review, we comprehensively described the EMT-inducing lncRNA molecular mechanisms and functional perspectives of EMT-inducing lncRNAs in GC progression. Taken together, the statements of this review provided a clinical implementation in identifying lncRNAs as potential therapeutic targets for advanced GC.

## Introduction

As the most frequently occurring malignancy worldwide, gastric cancer (GC) remains the fifth most diagnosed tumour and the third primary cause of tumour-related death (Sung et al., 2021). Approximately 1,089,103 people are diagnosed with GC worldwide each year, of whom about 783,000 die from this disease (Rawla and Barsouk, 2019; Yang L. et al., 2020). Asian countries, such as Japan, Mongolia, and Korea, show the highest incidence rates, with an estimated incidence rate per 100,000 of 48.1, 47.2, and 39.7, respectively (Morgan et al., 2022). GC is primarily divided into diffuse and intestinal types based on their histological characters (Lauren, 1965). Anatomically the two main types of GC are cardia and non-cardia subtypes. Additionally, the tumour, node and metastasis (TNM) system are used to assess tumour stage, including the tumour infiltration degree and size (T category), the lymph node status (N category), and the tumour distant metastasis to other organs (M category) (Amin et al., 2017). The important risk factors of the causes of GC are obesity (Kyrgiou et al., 2017), diabetes (Sona et al., 2018), smoking (Ladeiras-Lopes et al., 2008) and high salt intake (D'Elia et al., 2012). Although several risk factors are described, *Helicobacter pylori* infection-induced chronic inflammation is the most important known risk factor for GC (Huang et al., 1998; Uemura et al., 2001). The signs of GC in the early stage are vague and can remain undetected for years. Therefore, most GC patients are diagnosed late, often presenting tumour invasion or metastasis (Blum et al., 2013). In primary GC, the 5-year survival is 70%, whereas it is reduced to 32% amongst those with metastatic GC (Howlader N et al., 2020). In recent years, neoadjuvant chemotherapy, radiotherapy, and molecular-targeted regimen have become the mainstay of GC treatment (Digklia and Wagner, 2016; Song et al., 2017). Nevertheless, locally advanced, and metastatic GC patients still have a somber overall prognosis. Therefore, it is urgently necessary to further identify potential therapeutic targets to enhance the prognosis of patients with advanced GC.

Epithelial-mesenchymal transition (EMT) is a double-edged sword. EMT is a normal physiological process necessary for embryogenesis and wound healing. However, EMT dysregulation is a pathological process that result in cancer or fibrosis (Barriere et al., 2015; Yang J. et al., 2020). Gastrulation is an embryonic development of single layers embryo into three layers formation, of which the EMT is considered to be an important component. (Trelstad et al., 1967; Kim et al., 2017). EMT also provides a critical mechanism for the re-epithelialization of tissue which contributes to wound healing (Pastar et al., 2014). In addition, recent evidence has proven that the aberrant activation of EMT is closely linked with tumorigenesis, invasion, and metastasis of GC (Peng et al., 2014; Huang et al., 2015). As a complicated process, tumour metastasis mainly includes local invasion, intravasation, transport, and extravasation, by which malignant cells leave the primary tumour sites, sequentially colonize, and form secondary tumours at adjoining or distant organs, leading to GC-associated death (Yilmaz and Christofori, 2009). As a biological program, EMT triggers the detachment of polarized epithelial cells from neighbouring cells and converts them into mesenchymal cells, in which tumour cells forfeit epithelial polarity and transform into a mesenchymal phenotype, playing crucial roles in tumour invasion and metastasis (Kalluri and Weinberg, 2009). Epithelial markers (E-cadherin and α-catenin) and mesenchymal markers (N-cadherin, vimentin and fibronectin) are examined to determine whether cancer cells undergo the EMT (Zeisberg and Neilson, 2009). The GC patients with mesenchymal phenotype facilitate GC cell motility and metastasis and are correlated with advanced GC stage, while intestinal phenotype is mainly distributed in those at the early stage (Zheng et al., 2013). Accumulating evidence has revealed that the worst prognosis is associated with GC patients with EMT molecular subtype (Cristescu et al., 2015; Oh et al., 2018).

Non-coding RNAs (ncRNAs), including microRNAs (miRNAs), long non-coding RNAs (lncRNAs), and circular RNAs (circRNAs), can regulate the tumorigenesis of different tumours (Chan and Tay, 2018). Among these, lncRNAs dysregulation play an essential role in tumour metastasis and are conducive to the EMT (Bhan et al., 2017; Wei et al., 2020; Wang H. et al., 2021; Wang X. et al., 2021). Various lncRNAs are identified to enhance the migration, invasion of GC cells and promote the EMT process of GC (Gao et al., 2021; Li et al., 2022). Therefore, targeting lncRNAs has become a promising therapeutic regimen in patients with metastatic GC. In the present review, we summarised the regulatory functions of lncRNAs in the EMT-induced metastatic GC.

## The characteristics and mechanism of lncRNAs’ action in EMT-induced metastasis

The first transcript sequence of lncRNAs was discovered in eukaryotes. LncRNAs are longer than 200 nucleotides in length and cannot be translated into proteins, and their primary structure is nucleotide sequence. Most of the lncRNAs share some similar features with messenger RNAs (mRNAs) and can be spliced, capped and polyadenylated by RNA polymerase II (Sun et al., 2018). In the past, traditional gene annotation filtered out proteins with <100 amino acids and treated them as noise (Wu et al., 2020). In recent years, research has revealed that lncRNAs contain short open reading frames (sORFs) which encode functional small peptides of approximately 100 amino acids in length using proteomics and translation technology (Zhu et al., 2018; Choi et al., 2019). These micro peptides resemble coding and noncoding genes and function as tumour regulatory factors to get involved in angiogenesis, signalling pathway transduction and metabolism in promoting cancer progression (Wu et al., 2020; Ye et al., 2020).

The functional activity of lncRNAs includes regulating transcription of neighbouring and distant genes, affecting the stability of mRNAs and interfering with signalling pathways through their crosstalk with DNA, RNA and proteins (Statello et al., 2021). Based on genomic localization and transcriptional orientation, lncRNAs are divided into intergenic lncRNAs, intronic lncRNAs, exonic lncRNAs, sense lncRNAs, and antisense lncRNAs (Ma et al., 2013). Based on the subcellular localization, lncRNAs are classified as nuclear lncRNAs and cytoplasmic lncRNAs (Kapranov et al., 2007). Nuclear lncRNAs mainly mediate the transcription (Sun et al., 2018), and cytoplasmic lncRNAs commonly participate in post-transcriptional regulation and function as miRNA sponges (Du et al., 2016).

LncRNAs are a highly heterogeneous group of epigenetic regulators that can mediate EMT-induced metastasis utilizing diverse mechanisms. First, lncRNAs can regulate gene expression at chromatin modification, transcription processing, and post-transcriptional processing (Dykes and Emanueli, 2017). LncRNAs recruit chromatin remodelling complexes to specific sites and coordinate genome activity by controlling chromosome structure and affecting histone status and DNA methylation status (Beckedorff et al., 2013; Böhmdorfer and Wierzbicki, 2015), which is significantly associated with the overall suvival and disease-specific survival of GC (Dai J. et al., 2021). After transcription, most lncRNAs are transcribed by RNA polymerase II and share some similarities with mRNAs, including 5′ end capping, 3’ end polyadenylating, splicing, and intracellular transporting (Canzio et al., 2019). Second, transcription factors (TFs) play an essential role in the development of GC. LncRNAs have been implicated in gene transcription through biding to EMT-inducing TFs (Long et al., 2017). The lncRNAs - TFs interaction directly regulates gene transcription via inducing the targeted protein degradation by phosphorylation and ubiquitination (Lamouille et al., 2014; Xiu et al., 2019). Thirdly, some identified lncRNAs shows oncogenic properties, acting as competing endogenous RNAs (ceRNAs) to sponge miRNA at the post-transcriptional level, as consequently, the interaction with targeted mRNA, thus increasiong the expression of oncogenic mRNA to facilitate the cancer occurrence and progression (Arun K. et al., 2018; Liu H. et al., 2018; Qi et al., 2020).

## LncRNAs regulate EMT-induced metastasis by mediating the signalling pathways

EMT is induced by a variety of signalling pathways, including TGF-β, BMP, Wnt-β-catenin, NOTCH, Shh, and receptor tyrosine kinases, and many feedback activation/inhibition mechanisms have been demonstrated (Deshmukh et al., 2021). In the process above, lncRNAs regulate the EMT process in GC by mediating various signalling pathways, including Wnt, PI3K/AKT, Hippo, MEK/ERK, and Notch1 (Table 1).

**TABLE 1:** LncRNAs and their targeting signalling pathways in the regulation of EMT-induced GC metastasis.

Signalling pathway	lncRNA	Expression	Target	Function	References
Wnt/β-catenin	H19	Up	β-catenin	Promotes of H19 on GC cell EMT and metastasis	Liu et al. (2021)
	TTTY15	Up	Wnt1	Promotes proliferation, migration, invasion, EMT	Zheng et al. (2022)
	ZEB2-AS1	Up	ZEB2	Increases the proliferation, migration, invasion, and EMT and resistance to chemotherapeutic reagents	Wang et al. (2019a)
	LOC400043	Down	β-catenin	Impairs cell cycle, proliferation, and EMT ability and induces apoptosis	Jafarzadeh and Soltani, (2020)
	DLGAP1-AS2	Up	Six3, Wnt1	Improves the malignancy of GC	Lu et al. (2021)
	HOXC-AS1	Up	eIF4AIII	Promotes the proliferation and the EMT process and inhibit apoptosis	Chao et al. (2020)
	LINC01225	Up	—	promoted EMT and malignant progression of GC	Xu et al. (2019)
	ZFAS1	Up	NKD2	migration, invasion, EMT and resistance to chemotherapeutic reagents	Xu et al. (2018b)
GSK-3β/β-catenin	SNHG20	Up	EZH2	Promotes invasion capability and EMT in the GC	Liu et al. (2017)
PI3K/AKT	TM4SF1-AS1	Up	M4SF1	Promotes cancer cell proliferation, invasion and the EMT	He et al. (2021a)
	TNK2- AS1	Up	miR-125a-5p	Promotes the malignant behaviours of GC cells AGS	Guo et al. (2022)
PI3K/AKT/mTOR	XLOC_006753	Up	—	Promotes MDR GC cell migration through enhancing EMT	Zeng et al. (2018)
TGF-β	LINC00665	Up	—	Promotes GC cell proliferation, invasion, and metastasis	Zhang and Wu, (2021)
	SGO1-AS1	Down	PTBP1	Prevents the EMT and metastasis	Huang et al. (2021)
MEK/ERK	AL139002.1	Up	miR-490-3p/HAVCR1	Regulates EMT and metastasis	Chen and Zhang, (2021)
Hippo	LincRNA-p21	Down	YAP	knockdown correlates with higher invasion depth grade and induces EMT	Ying et al. (2017a)
Notch1	SNHG1	Up	DCLK1	Enhances the EMT process in GC cells	Liu et al. (2020a)

Wnt is a secretory glycoprotein that acts by autocrine or paracrine. After secretion, Wnt can bind to cell surface-specific receptors, leading to β-catenin accumulation *via* phosphorylating and dephosphorylating a series of downstream proteins. As a multifunctional protein, β-catenin reacts with E-cadherin at the cell junctions and is involved in the formation of adhesive bonds. Free β-catenin can reach the nucleus and get involved in gene expression, while its dysfunction or activation can trigger tumorigenesis. The primary elements of the Wnt signalling pathway consist of secreted protein Wnt family, transmembrane receptor Frizzled family, casein kinase (CK1), Dishevelled (DVL), adenomatous polyposis coli (APC), Axin, glycogen synthase kinase 3-beta (GSK3β), β-catenin and TF transcription factor T-cell factor/lymphoid enhancer-binding factor (TCF/LEF) family (MacDonald et al., 2009; Zhan et al., 2017; Albrecht et al., 2021).

LncRNA H19 is increased in different malignancies, and it functions as an oncogene. H19 is overexpressed in GC and associated with poor prognosis. H19 can transfer β-catenin into the nucleus and activate Wnt/β-catenin signalling, facilitating EMT of GC cell (Liu et al., 2021). LncRNA ViM antisense RNA 1 (VIM-AS1) is highly expressed in GC and associated with prognostic outcomes. VIM-AS1 may enhance cell migration, invasion, and EMT by mediating frizzled 1(FDZ1), and activating the Wnt/β-catenin pathway (Sun et al., 2020). The expression of lncRNA Zinc finger E-box-binding homeobox two antisense RNA 1 (ZEB2-AS1) is up-regulated in GC specimens, down-regulated of ZEB2-AS1 can suppress the proliferation, EMT, and Wnt/β-catenin signalling (Wang F. et al., 2019). LncRNA testis-specific transcript, Y-linked 15 (TTTY15) (Zheng et al., 2022), lncRNA DLGAP1 antisense RNA 2 (DLGAP1-AS2) (Lu et al., 2021), and lncRNA HOXC cluster antisense RNA 1(HOXC-AS1) (Zhou C. et al., 2020) are also overexpressed and can regulate the Wnt/β-catenin signalling pathway to promote EMT in GC.

GSK-3β is a serine/threonine-protein kinase. In the absence of Wnt signalling, phosphate groups can be added to n-terminal serine/threonine residues of β-catenin by GSK-3β. After covalent modification by β-TRCP ubiquitination, the phosphorylated β-catenin is degraded (Stamos and Weis, 2013). LncRNA small nucleolar RNA host gene 20 (SNHG20) acts as an oncogene in GC. The expressions of E-cadherin and p21 can be markedly suppressed when SNHG20 is overexpressed in MKN45 and BGC-823 cells *via* binding to the enhancer of zeste homolog 2 (EZH2) and mediating the GSK-3β/β-catenin signalling pathway, and SNHG20 can be a therapeutic target for GC (Liu et al., 2017).

The AKT, also called protein kinase B, signalling pathway is involved in the molecular mechanisms underlying many cancers, which plays a vital role in tumor cell proliferation, metastasis and drug resistance, lncRNAs can regulate the relative expressions of key genes in the phosphoinositide 3-kinase (PI3K)/AKT pathway (Peng et al., 2017; Lin et al., 2020). The PI3K/Akt signalling pathway can impact the EMT in various manners to alter the aggressiveness of cancer (Xu et al., 2015). GC tissues and cells exhibit increased expression of lncRNA Transmembrane four superfamily 1-antisense 1 (TM4SF1-AS1). However, the proliferation, invasion, EMT and enhanced apoptosis of cancer cells can be inhibited when TM4SF1-AS1 is depleted. The underlying mechanism is associated with the suppression of TM4SF1 and PI3K-AKT signalling pathways (He C. et al., 2021).

Transforming growth factor-β (TGF-β) family is a group of structurally related proteins, is involved many cellular functions, including EMT and migration, and many human diseases, including vascular diseases, autoimmune disorders, and carcinogenesis (Syed, 2016). LncRNA Shugoshin-like protein 1-antisense 1 (SGO1-AS1) facilitates TGF-β1/2 mRNA decay by competitively binding to the PTBP1 protein, leading to impaired TGF-β production, and preventing EMT and metastasis (Huang et al., 2021). Extracellular signal-regulated kinase (ERK) cascade can regulates proliferation, differentiation, survival, and apoptosis of cells, the mitogen extracellular signal-regulated kinase (MEK) functions as an upstream essential protein of ERK (Liu W. et al., 2015). LncRNA AL139002.1 is highly expressed in GC cells, and lncRNA AL139002.1/miR-490-3p/HAVCR1 functions critically in GC by mediating the MEK/ERK signalling (Chen and Zhang, 2021). The Hippo pathway can mainly restrict adult tissue growth and regulate cell proliferation, differentiation, and migration in developing organs. In addition, abnormal cell growth and neoplasia are observed when the Hippo pathway is dysregulated (Meng et al., 2016). LINC00649 acts as an oncogene to promote the EMT by targeting the miR-16-5p/YES-associated protein 1 (YAP1)/Hippo signalling pathway (Wang H. et al., 2021).

The Notch family consists of four highly conserved transmembrane receptors. Enzyme activity of G-secretase is required to release active regions within cells. Notch is involved in many physiological processes of embryonic development and normal cells, regulating cell growth, apoptosis, and differentiation. Notch1, a member of the Notch family, has been linked to various cancers (Gharaibeh et al., 2020). LncRNA small nucleolar RNA host gene 1(SNHG1) is overexpressed in GC and regulates the EMT process and cell migration by miR-15b/DCLK1/Notch1 axis (Liu Z. Q. et al., 2020).

## LncRNAs regulate EMT-induced metastasis in GC through transcription factors

TFs also known as DNA-binding factors, control the transcription from DNA to RNA *via* binding to a specific DNA sequence (Latchman, 1993). TFs regulates RNA polymerase activity *via* interacting with two classes of surface domain: a sequence-specific DNA binding domain (i.e., zinc finger and homeodomain) and an activation domain that binds to various cofactors to recruit RNA polymerase (Bhagwat and Vakoc, 2015; Mulero et al., 2018). LncRNAs exist in both cytoplasm and nucleus and their functions are activated by two main mechanisms. In the nucleus, lncRNAs bind to TFs directly by interacting with DNA to regulate the transcription of GC metastasis-related genes (Fatima et al., 2015). In the cytoplasm, lncRNAs bind to tissue-specific protein, altering the post-translational modification to induce the protein ubiquitination and degradation (Table 2) (Liao et al., 2021).

**TABLE 2:** LncRNAs and their associated transcription factors in the regulation of EMT-induced metastasis in gastric cancer.

LncRNA	Expression	EMT-related targets	Biological functions	References
AC093818.1	Up	PDK1	Accelerates GC tumour metastasis	Ba et al. (2020)
AGAP2-AS1	Up	SP1	Increases GC cell migration and invasion	Qi et al. (2017)
AK023391	Up	c-Myb and BCL-6	Increases GC cell invasion *in vitro* and GC tumour metastasis *in vivo*	Huang et al. (2017)
CASC2	Down	E2F6	Inhibits GC cells invasion	Li et al. (2019)
CASC15	Up	ZEB1	Increases GC tumour volume and weight *in vivo*	Wu et al. (2018)
DANCR	Up	SALL4	Increases GC cells migration and invasion	Pan et al. (2018)
DLGAP1-AS2	Up	SLUG and TWIST	Increases GC cell AGS migration and invasion	Lu et al. (2020b)
DLX6-AS1	Up	OCT1	Increases GC cell migration, invasion and EMT	Liang et al. (2020)
GAPLINC	Up	HIF-1α	Promotes GC tumour invasion behaviour	Liu et al. (2016b)
H19	Up	RUNX1	Increases GC cell AGS invasion	Liu et al. (2016a)
HOTTIP	Up	HMGA1	Increases GC cell migration and invasion	Wang et al. (2019b)
Lnc01614	Up	SNAIL	Increases GC cell migration and invasion	Dong et al. (2018)
LINC00261	Down	SLUG	Promotes lung metastasis of GC *in vivo*	Yu et al. (2017)
LINC01272	Up	ZEB2, TWIST	Increases GC cell migration and invasion	Leng et al. (2020)
LINC-ROR	Up	OCT4, SOX2 and NANOG	Increases invasion of GC	Wang et al. (2016b)
LincRNA-p21	Down	YAP	Promotes malignant behaviour of lincRNA-p21 knockdown GC cells	Chen et al. (2017)
LncRNA-AF147447	Down	E2F1	Inhibits GC cell migration and invasion *in vitro* and *in vivo*	Zhou et al. (2016)
LOXL1-AS1	Up	USF1	Increases GC cell migration and EMT	Sun et al. (2019a)
MAG12-AS3	Up	ZEB1/2	Increases GC cell migration and invasion	Li et al. (2020a)
MALAT1	Up	SNAIL	Increases GC cell AGS migration and invasion	Lee et al. (2017)
MALAT1	Up	SNAIL	Enhances GC tumour invasion	Lee et al. (2015)
MIR99AHG	Up	FOXP1	Increases GC cell migration and invasion	Meng et al. (2020)
MNX1-AS1	Up	TEAD4	Increase GC cell migration and invasion	Shuai et al. (2020)
PCGEM1	Up	SNAI1	Increases GC cell invasion and metastasis	Zhang et al. (2019a)
PVT1	Up	FOXM-1	Increases GC cell invasion *in vitro* and *in vivo*	Xu et al. (2017)
RGMA-AS1	Up	NFIB	Increases GC cell migration and invasion	Zhang et al. (2020a)
SEMA3B-AS1	Down	Sp1	Inhibits GC cell migration and invasion	Guo et al. (2019b)
SNHG20	Up	TWIST	Increases expression level of Twist expression in GC cell MKN45	Liu et al. (2017)
TRERNA1	Up	SNAI1	Increases GC cell migration and invasion and GC tumour metastasis	Wu et al. (2017)
UCA1	Up	ZEB2	Increases GC cell migration and invasion	Gong et al. (2018)

LncRNAs participate in tumour progression and metastasis by modulating EMT (Xu et al., 2016). In addition, lncRNAs function critically in the induction and regulation of EMT-TFs (Pavlič et al., 2022). The loss of expressions of the cadherin family proteins remains the hallmark of EMT, which is crucial in cell-cell adherents junctions (Wang and Zhou, 2013). During EMT, decreased expression of E-cadherin translocate β-catenin to the nucleus and activates numerous notable TFs, including SNAIL, SLUG, Twist-related protein 1 (TWIST1), zinc-finger E-box-binding homeobox 1 (ZEB1), and 2 (ZEB2) (Chan and Wang, 2015; Stemmler et al., 2019).

SNAIL (also known as SNAI1), a zinc-finger transcriptional repressor, modulates EMT during tumour progression (Wang et al., 2013). It binds to E-box, an E-cadherin promoter region, which converts epithelial cells to mesenchymal cells (Villarejo et al., 2014). The overexpression of SNAIL up-regulates XBP1 (Li et al., 2015) and ALX1 (Yuan et al., 2013), subsequently inducing the activation of EMT. The Notch activity (Timmerman et al., 2004) and Wilms’ tumour one homolog (Wt1) (Martínez-Estrada et al., 2010) promote EMT through transcriptional induction of the SNAIL repressor. Additionally, overexpression of SNAIL suppresses miR-192 and miR-194 but up-regulates miR-205, let-7i, and SNORD13. Those identified changes are correlated with the initiation of SNAIL-mediated EMT in cancer cells (Przygodzka et al., 2019). Depleting lnc01614 in GC cells (SGC7901 and AGS) exhibits attenuated migration and invasion caused by decreased SNAIL expression (Dong et al., 2018). Lee and their colleagues have also confirmed that siR-MALAT1 reduces gastric tumorigenesis by inhibiting invasiveness *via* reduced expression of SNAIL (Lee et al., 2017). The lncRNAs TRERNA and PCGEM1 function as enhancers of SNAI1 to contribute to the metastasis of GC (Zhang J. et al., 2019).

In addition to SNAIL, SLUG is another notable SNAIL superfamily of zinc-finger TFs. It acts as a transcriptional repressor to bind to E-box to mediate the expressions of target genes responsible for the EMT (Stegmann et al., 1999). The expression of SLUG is controlled by Tbx18 and Wt1, which regulate EMT activation (Takeichi et al., 2013). Moreover, the high-mobility group AT-hook 2 (HMGA2) has a positive correlation with SLUG expression in EMT activation (Li et al., 2014). The work performed by Lu and their colleagues has demonstrated that depletion of DLGAP1-AS2 suppresses the migration and invasion of GC cells AGS *via* down-regulating SLUG (Lu J. et al., 2020).

As a primary helix-loop-helix TF, TWIST participates in recognizing E-box elements. Overexpression of TWIST plays an essential role in promoting EMT (Chava et al., 2019). TWIST activation is controlled by several signalling pathways, such as Akt (Tang et al., 2016) and STAT3 (Zhang et al., 2015). Overexpression of TWIST up-regulates miR-214 to facilitate the EMT process (Liu C. et al., 2018). The *in vitro* investigation has indicated that inhibition of LINC01272 and DLGAP1-AS2 attenuates the migration and invasion of GC cells. Exposure of GC cells to LINC01272 siRNA and DLGAP1-AS2 siRNA significantly inhibits the expression of TWIST (Lu J. et al., 2020; Leng et al., 2020).

ZEB1/2 encodes zinc finger and down-regulates E-cadherin to induce EMT in carcinomas (Bürglin and Affolter, 2016). Li and their colleagues have confirmed that the expression of ZEB1/2 is significantly inhibited in MAG12-AS3-depleted GC cells (Li D. et al., 2020). In another case, lncRNA UCA1 contributed to GC metastasis *via* regulating miR-203/ZEB2 axis (Gong et al., 2018).

In addition to classic EMT-TFs described above, lncRNAs also regulate some other TFs to influence the progression and metastasis of GC. For example, overexpression of forkhead box M1 (FOXM1) enhances GC cell motility, and this effect can be reversed by blocking Cath-D (Yang et al., 2017). Cytoplasmic lncRNA plasmacytoma variant translocation 1 (PVT1) is a valuable prognostic predictor in GC. High PVT1 expression promotes the invasiveness of GC cell lines through binding to FOXM1 protein which implicate in high TNM staging and lymph node metastasis (Xu et al., 2017). The lncRNA MNX1-AS1 enhances the migration and invasion of GC cells *in vitro*. TEA domain DNA-binding family of TF 4 (TEAD4) acts as an oncogene to mediate Hippo signalling driving cancer progression. By binding to TEAD4, MNX1-AS1 promotes tumorigenesis through up-regulating BCL2 expression (Shuai et al., 2020). Additionally, the study performed by Zhou *et al.* have shown that the EMT-induced lncRNA AF147447 negatively regulates the expression of E2F1 and promotes GC tumour metastasis *via* the miR-34c/MUC2 axis (Zhou et al., 2016).

## LncRNAs regulate EMT-induced metastasis in GC through sponging miRNAs

miRNAs are a group of small RNAs with approximately 22 nt in length. miRNAs bind to the complementary sequence in targeted mRNAs, leading to the degradation of targeted mRNAs *via* RNA-induced silencing complex (RISC). Like lncRNAs, miRNAs play a critical role in different tumours. Many miRNAs are significantly up-regulated in cancer cells, resulting in cancer development. Several miRNAs even mediate the progression of various tumours (Karagkouni et al., 2021). In the study on the regulation of gene expression, miRNA and lncRNA are vital links. One of the widely recognized types is the endogenous competition mechanism. Different from directly regulating target genes, some lncRNAs inhibit the degradation or inhibition effect of miRNAs on target genes by binding to miRNAs. Such a regulatory strategy is widely reported in GC. LncRNA metallothionein 1 J, pseudogene (MT1JP) sponges miR-92a-3p and mediates the downstream F-Box-WD Repeat-Containing Protein 7(FBXW7) gene, which in turn impacts the progression of GC (Zhang G. et al., 2018). LINC01234 functions as the ceRNA of miR-204-5p and blocks the activation of core-binding factor b (CBFB) in GC (Chen et al., 2018). Some lncRNAs that regulate the target genes and are involved in the EMT progress of GC *via* the competitive binding of lncRNAs and miRNAs are summarized in Table 3.

**TABLE 3:** LncRNAs and their associated miRNAs in the regulation of EMT-induced metastasis in GC.

LncRNA	Expression	miRNA	Biological function	References
ACTA2-AS1	Down	miR-378a-3p/PLCXD2	Inhibits GC cell viability, migration, invasion and EMT process	Liu et al. (2022)
ASNR	Up	miR-519e-5p/FGFR2	Promotes EMT	Chen et al. (2021b)
CCL2	Up	miR-128/PARP2	Promotes migration, invasion and EMT	Liang et al. (2022)
DLX6-AS1	Up	miR-204-5p/OCT1	Promotes GC progression and the EMT process	Liang et al. (2020)
FAM225A	Up	miR-206/ADAM12	Promotes the development of GC and EMT	Chen et al. (2021a)
H19	Up	miR-152-3p/TCF4	Promotes EMT process	Jiang et al. (2020)
HCP5	Up	miR-186-5p/WNT5A	Promotes EMT	Gao et al. (2021)
HCG18	Up	miR-152-3p/DNAJB12	Promotes GC progression and EMT	Ma et al. (2020)
HNF1A-AS1	Up	miR-30b-5p/EIF5A2	Promotes EMT process	Jiang et al. (2022)
HOTTIP	Up	miR-218/HMGA1	Promotes migration, invasion, and EMT process	Wang et al. (2019b)
HOTAIR	Up	miR-217/GPC5	Promotes GC development, invasion and EMT process	Dong et al. (2019)
LINC01050	Up	miR-7161-3p/SPZ1	Contributing to GC progression and promotes EMT	Ji et al. (2021)
LINC00240	Up	miR-124-3p/DNMT3B	Promotes GC cell proliferation, migration and EMT	Li et al. (2020d)
LINC00689	Up	miR-526b-3p/ADAM9	Promotes the proliferation, migration, invasion and EMT of GC cells	Yin et al. (2020)
LINC00649	Up	miR-16-5p/YAP1	Promotes cell proliferation, migration and EMT in GC.	Wang et al. (2021a)
LINC00689	Up	miR-338-3p/HOXA3	Increases EMT development	Lu et al. (2020a)
LINC01133	Down	miR-106a-3p/APC	Inhibits proliferation, migration and EMT of GC cells	Yang et al. (2018)
MAG12-AS3	Up	miR-141/200a-3p/HMGB2	Increases GC cell migration, invasion and promotes EMT process	Li et al. (2020a)
MALAT1	Up	miR-1297/HMGB2	Promotes cell proliferation, invasion and EMT process in GC	Li et al. (2017)
MIAT	Up	miR-331-3p/RAB5B	promoted proliferation and metastasis, and inhibited the apoptosis of GC cells.	Li et al. (2020c)
MIR99AHG	Up	miR577/FOXP1	Promotes GC progression by inducing EMT process	Meng et al. (2020)
MIR503HG	Down	miR-224-5p/TUSC3	Represses EMT process and GC progression	Lin et al. (2021)
NR2F1-AS1	Up	miR-190a/PHLDB2	Promotes the phosphorylation of AKT3 to induce EMT in GC cells	Lv et al. (2021)
PCED1B-AS1	Up	miR-215-3p/CXCR1	Promotes EMT	Ren et al. (2021)
PCAT6	Up	microRNA-30/MKRN3	Promotes EMT	Xu et al. (2018c)
RGMB-AS1	Up	miR-22-3p/NFIB	Accelerates the progression of EMT and GC	Zhang et al. (2020a)
SNHG6	Up	miR-101-3p/ZEB1	Promotes cell proliferation and EMT	Yan et al. (2017)
SNHG6	Up	miR-1297/BCL-2	Promotes GC tumour growth and EMT process	Mei et al. (2021)
SNHG7	Up	miR-34a/Snail	Promotes EMT initiation to enhances GC cell migration and invasion	Zhang et al. (2020b)
SNHG1	Up	miR-140/ADAM10	Promotes GC cell invasion and EMT	Guo et al. (2019a)
SNHG3	Up	miR-326/TWIST	Promotes metastasis by inducing EMT	Rao et al. (2021)
TMPO-AS1	Up	miR-140-5p/SOX4	Promotes cell migration and invasion and EMT process	Sun and Han, (2020)
UBE2CP3	Up	miR-138-5p/ITGA2	promotes EMT signalling	Li et al. (2021)

Some miRNAs can regulate the expressions of TFs, while lncRNAs can suppress the degradation of target genes by binding these miRNAs to promote the expressions of TFs, leading to the promoted EMT process of GC. SNHG1 promotes the proliferation and invasion of GC cells *via* modulating the miR-140/ADAM10 axis (Guo et al., 2019a). GC cell lines display markedly high expressions of lncRNAs small nucleolar RNA host gene (SNHG3) and TWIST. Depletion of SNHG3 significantly inhibits the proliferation, migration, and invasion of GC cell lines. SNHG3 acts as an endogenous sponge to reduce the expression of miR-326 and regulates the expression of TWIST by competitively binding to miR-326 (Rao et al., 2021). Overexpression of small nucleolar RNA host gene 7 (SNHG7) has been reported in most human tumors, including lung cancer, and it acts as an oncogenic lncRNA in GC and may be a promising therapeutic candidate for GC patients. SNHG7 promotes the migration and invasion of GC cells by inhibiting miR-34a (Zhang Y. et al., 2020). As a potential oncogene, small nucleolar RNA host gene 6 (SNHG6) is involved in the initiation and progression of hepatocellular carcinoma, and SNHG6 functions as an oncogene in GC cells by post-transcriptionally mediating and transcriptionally silencing miR-101-3p/ZEB1 *via* recruiting EZH2 to the promoter of p27 (Yan et al., 2017).

Emerging evidence has shown that EMT plays a critical role in the chemoresistance of tumor cells. The resistance of lung cancer cells to doxorubicin can be effectively reversed by inhibiting EMT (Ying Y. et al., 2017). EMT is associated with treatment resistance (Gaianigo et al., 2017), suppressing EMT may enhance the chemosensitivity. SNHG6 positively regulates B-Cell Lymphoma 2 (BCL-2) by sponging miR-1297. The DDP resistance, proliferation, and metastasis of DDP-resistant cells can be suppressed by depletion of SNHG6 (Mei et al., 2021). Exosome HOTTIP can regulate the miR-218/high-mobility group A1 (HMGA1) axis, contributing to cisplatin resistance in GC cells. HOTTIP regulates HMGA1 by acting as a ceRNA of miR-218 in GC cells. Serum exosome HOTTIP has been related to cisplatin resistance in GC patients (Wang et al., 2019b), lncRNA H19 suppresses the chemosensitivity to ADM *via* sponging miR-152 from TCF4 in GC cells (Jiang et al., 2020). HNF1A-AS1 promoted chemoresistance by facilitating EMT process through upregulating EIF5A2 expression by sponging of miR-30b-5p (Jiang et al., 2022).

LncASNR (apoptosis suppressing-non-coding RNA) inhibits the expression of miR-519e-5p but up-regulates fibroblast growth factor receptor 2 (FGFR2). As a receptor for FGF, FGFR2 can deliver the FGF signal to RAS-ERK and PI3K-AKT signal cascades, facilitating EMT-related migration and invasion of GC cells (Chen Z. et al., 2021). Overexpression of lncRNA HLA complex group 18 (HCG18) induced by hepatocyte nuclear factor 1 homeobox A (HNF1A) promotes GC progression by competitively binding to miR-152-3p and up-regulating DNAJB12. HNF1A can facilitate its transcription by binding to the HCG18 promoter. The DNAJB12 and cytosolic heat shock protein 70 (Hsp70) can promote the triage of nascent polytopic membrane proteins for folding or degradation by cooperating on the endoplasmic reticulum’s cytoplasmic face (Ma et al., 2020). GC tissues and cell lines show high expression of lncRNA PCED1B antisense RNA 1 (PCED1B-AS1), and its expression has been linked to the clinicopathological characteristics of GC patients. Moreover, PCED1B-AS1, as a ceRNA, up-regulates C-X-C motif chemokine receptor 1 (CXCR1) by competitively binding to miR-215-3p, leading to enhanced malignancy of GC cells, and this finding indicates that PCED1B-AS1/miR-215-3p/CXCR1 axis may be a potential mechanism involved in the progression of GC (Ren et al., 2021). CXCR1 can regulate the malignant biological behaviors of cancer cells by controlling the activation of AKT and ERK1/2 signaling pathways. Depletion of CXCR1 up-regulates E-cadherin in GC cells (Wang J. et al., 2016). Nuclear receptor subfamily two group F member 1-antisense RNA 1 (NR2F1-AS1)/miR-190a/Pleckstrin Homology Like Domain Family Member 2 (PHLDB2), a ceRNA, can facilitate the EMT process of GC cells, and PHLDB2 can enhance the expression and phosphorylation of AKT3 to promote the EMT process of GC cells (Lv et al., 2021). LINC00689/miR-526b-3p/A disintegrin and metalloproteinase domain 9 (ADAM9) participates in many biological processes, including myogenesis, fertilization, cell migration, inflammatory response, proliferation, and cell-cell interactions (Yin et al., 2020). miR-338-3p has a negative correlation with LINC00689 in GC. Homeobox A3 (HOXA3) is one target gene of miR-338-3p, and ectopic expression of LINC00689 inhibits miR-338-3p and up-regulates HOXA3 in GC cells (Lu H. et al., 2020). HOXA3 can activate EGFR/Ras/Raf/MEK/ERK signalling pathway, promoting the tumor growth of colon cancer. LINC00689 functions as a ceRNA by sponging miR-526b-3p in GC cells (Zhang X. et al., 2018).

As growth factors, cell signal transducers, and nuclear TFs, proto-oncogenes primarily regulate biological activities in normal cells. Changes in these genes affect their encoded proteins, becoming oncogenes, which drive cell proliferation and play a critical role in tumorigenesis (Kontomanolis et al., 2020). The expression of HOTAIR is often increased in GC tissues and cell lines, and a high expression of HOTAIR has been linked with poor prognosis in GC patients. HOTAIR can sponge miR-217 and inhibit its expression in GC. HOTAIR can facilitate the development of GC by up-regulating glypican-5 (GCP5) *via* sponging miR-217 (Dong et al., 2019). GPC5 is an oncogene and may play a critical role in regulating tumorigenesis. Early studies have confirmed that miR-217 functions as a cancer suppressor by directly targeting the GPC5 oncogene in GC (Wang et al., 2015). HOTAIR can interact with polycomb repressive complex 2 (PRC2), thereby mediating the downstream targets *via* epigenetic regulation. HOTAIR also binds to PRC2 to activate its target genes C-Met (HGF/C-Met/Snail pathway) and Snail *via* epigenetically decreasing the expression of miR34a, thereby facilitating EMT in advanced stages of GC (Liu Y. W. et al., 2015).

Depletion of LINC00649 inhibits YAP1 and releases miR-16-5p, leading to the recovery of the Hippo pathway, and some downstream oncogenes are suppressed accordingly, such as EGFR, SOX2, and OCT4, suppressing the malignant phenotypes in GC cells (Wang H. et al., 2021). YAP1 has been identified as an oncogene, which can promote the pathogenesis of multiple cancers and immunosuppression (He S. et al., 2021; Wang and Gao, 2021). LncRNA metastasis associated lung adenocarcinoma transcript 1 (MALAT1) promotes cell proliferation and invasion in GC, and its up-regulation is associated with local invasion, lymph node metastasis, and TNM stage. MALAT1 is negatively correlated with miR-1297 and functions as a molecule sponging miR-1297, antagonizing its ability to inhibit the expression of high mobility group box 2 (HMGB2) (Li et al., 2017). HMGB2 is an essential protein in carcinogenesis, and it is associated with increased proliferation, invasion, and glycolysis of GC cells (Cui et al., 2019). FAM225A (Chen N. et al., 2021), HCP5 (Gao et al., 2021), MIAT (Li X. M. et al., 2020), and UBE2CP3 (Li et al., 2021) all can promote the expressions of oncogenes.

In normal cells, there are tumor suppressor genes besides oncogenes. Tumor suppressor genes play a fundamental role in the normal growth and differentiation of cells. They protect the body from tumor invasion, block tumor growth, and promote the normal development of cells (Kontomanolis et al., 2020). LncRNA actin alpha 2, smooth muscle antisense RNA 1 (ACTA2-AS1) can suppress malignant phenotypes of GC cells as it can function as a ceRNA to bind to miR-378a-3p and antagonize the inhibitory impacts of miR-378a-3p on the expression of phosphatidylinositol-specific phospholipase C X domain containing 2 (PLCXD2) (Liu et al., 2022). PLCXD2 is linked to an enhanced risk of esophageal squamous cell carcinoma in the Han Chinese population (Wang et al., 2019c). LncRNA MRI503HG up-regulates tumour suppressor candidate 3 (TUSC3) in GC cells through sponging miR-224-5p, leading to GC progression. Depletion of ATF6 partially rescues EMT in GC cells overexpressing lncRNA MIR503HG. GC cell invasion is inhibited by overexpressing lncRNA MIR503HG, which reduces protein contents of N-cadherin and vimentin to hinder EMT in GC cells (Lin et al., 2021). Overexpression of TUSC3 impedes cell proliferation and triggers apoptosis in retinoblastoma (Kong et al., 2020).

As an essential epigenetic regulation, methylation can be described as the transfer of the active methyl group to the target chemicals catalyzed by methyltransferases, and the DNA sequence composition remains unchanged in this process. Methylation deregulation is involved in many diseases, including human cancers (Dai X. et al., 2021). Ubiquitination regulates several steps in autophagy *via* post-translational modification, which is a primary lysosome-mediated intracellular degradation pathway. Multiple ubiquitin chains act as selective markers to attach to protein aggregates and dysfunctional organelles, thereby accelerating the degradation in an autophagy-dependent manner (Grumati and Dikic, 2018). Overexpression of lncRNA prostate cancer-associated transcript 6(PCAT6) has been reported in GC, which facilitates the progression of GC by endogenous competition with miRNA-30 by targeting makorin ring finger protein 3 (MKRN3). MKRN3 participates in the processes of gene transcription and ubiquitination. The invasive ability of GC cells is enhanced when PCAT6 is overexpressed. The expressions of EMT-related genes at the protein level are also remarkably increased (Xu Y. et al., 2018). LncRNA The C-C motif chemokine ligand 2 (CCL2) suppresses the expression of miR-128 in GC. miR-128 mimic significantly down-regulates the expression of poly (ADP-ribose) polymerase 2 (PARP2). As a leading member of the PARP family, PARP2 possesses multiple biological functions, such as DNA repair, synthetic lethality, apoptosis, necrosis, and histone binding (Ma et al., 2022). LINC00240 can bind to miR-124-3p as a ceRNA to enhance the expression of DNA methyltransferase 3b (DNMT3B), a member of the DNMT family. DNMT3B can enhance cell proliferation, invasion, and migration of GC cells. siRNA targeting LINC00240 up-regulates E-cadherin and down-regulates vimentin in GC SGC-7901 cells and BGC-823 cells (Li Y. et al., 2020).

In addition to the widely recognized endogenous competition mechanisms, there are a few other mechanisms of lncRNAs and miRNAs. For example, miR-21 is negatively mediated by lncRNA Maternally expressed gene 3 (MEG3) and can enhance metastasis in GC. The MEG3/miR-21 axis is involved in the progression and metastasis of GC through mediating EMT (Xu G. et al., 2018).

## Diagnostic and prognostic value of EMT-related LncRNAs in metastatic gastric cancer

Most GC--associated mortality is attributed to tissue metastasis. GC preferably metastasizes to the liver accounting for 48% of metastatic GC patients. Moreover, the other common sites for GC to spread include the peritoneum, lung and bone, accounting for 32%, 15% and 12% of patients with metastatic cancer, respectively (Riihimäki et al., 2016). At present, the combined chemotherapy protocols, such as FOLFOX (oxaliplatin and 5-FU/leucovorin), CAPOX (oxaliplatin and capecitabine) and FOLFIRI (irinotecan and 5-FU/leucovorin) are the most commonly regimen of chemotherapy treatment for GC. In addition, targeted drugs (Trastuzumab, Ramucirumab, Larotrectinib and Entrectinib) might be helpful in GC patients with gene over-expression and mutation. Growing evidence has shown that immunotherapy is a promising treatment for GC. FDA approved nivolumab and pembrolizumab, in combination with chemotherapy, for the treatment of patients with locally advanced or metastatic GC (Takei et al., 2022). Although the availability of numerous drugs for the treatment of GC, 39% of GC patients were found to have metastatic disease and had a poor prognosis (Dai W. et al., 2021). Thus, there is an urgent need to find potential valuable biomarkers for prognosis of patients with metastatic GC.

Carbohydrate antigen (CA) 12-5 and CA 72-4 are the most frequently used biomarkers in diagnosis of patients with GC (Matsuoka and Yashiro, 2018). In addition, carcinoembryonic antigen (CEA) and CA 19-9 act as the prognostic predictors in clinical practice, as they have not detected in the early stage of GC (Feng et al., 2017). Therefore, some novel reliable markers supporting diagnosis and prognosis of GC are needed. Previous study has demonstrated that circulating lncRNAs is detectable in plasma, and it significant increase in GC patients than that of health donors (Arita et al., 2013). Therefore, lncRNAs are adopted to diagnose tumors at early stages, and they can also predict the prognosis, metastasis risks, and recurrence after surgery (Necula et al., 2019).

As diagnostic biomarkers, some lncRNAs are differentially expressed in the serum and plasma of GC patients and normal patients. For example, using gastric juice ABHD11-AS1 as a marker, ABHD11-AS1 levels were significantly increase in early GC patients, reaching to 71.4% (Yang Y. et al., 2016). Serum B3GALT5-AS1 levels were significantly higher in GC patients than that of in normal individuals. High serum B3GALT5-AS1 levels were also associated with TNM stage and lymph node metastasis (Feng et al., 2020). The level of serum exosome lncRNA H19 in GC patients was significantly up-regulated before and after surgery when compared with that in healthy controls, and the postoperative level was significantly lower than that before operation. Preoperative lncRNA H19 levels were significantly correlated with TNM stage. The area under the ROC curve (AUC) value of exosome lncRNA H19 was significantly higher than the AUC value of cancer antigen 19-9, 72-4 and carcinoembryonic antigen (Feng et al., 2020). The AUC of exosome HOTTIP was 0.827, and its diagnostic ability was significantly higher than that of CEA, CA 19-9 and CA72-4 and exosome HOTTIP overexpression was an independent prognostic factor in GC patients (Feng et al., 2020). In addition, several studies have investigated the effects of *H. pylori* infection on GC progression by regulating lncRNAs expression. For example, lncRNA AF147447 decreased expression by *H. pylori* infection and acts as a tumour suppressor in the development of GC (Zhou et al., 2016). Li and their collegues addressed a significant associations with high expression of lncRNA51663 and FLJ46906 and increased risk of *H. pylori* infection-induced GC (Li N. et al., 2020). In addition, serum H19 and LINC00152 could function as potential biomarkers for GC patients with *H. pylori* infection due to the joint effect of H19 and LINC00152 and *H. pylori* infection on the increased risk of GC (Yang T. et al., 2016).

Accumulating evidence has shown that lncRNAs can act as prognostic biomarkers in predicting GC tumor size and Lauren classification, depth of invasion, Lymph node and distant metastasis, TNM stage. Highly expressed lncRNA DANCR was tested in tumour tissues and serum form GC patients than that of from healthy controls (Pan et al., 2018). Moreover, HOTAIR expression is significantly elevated in GC tissues when compared with adjacent non-cancer tissues. This study also confirmed the association of HOTAIR overexpression with poor overall survival in patients with diffuse-type GC (Petkevicius et al., 2022). Yang and their colleagues have performed study to determine the expression of lncRNA ABHD11-AS1 in gastric juice from GC patients relate to tumour size, tumour stage, Lauren type and blood CEA level (Yang Y. et al., 2016). In addition, elevated expression of lncRNA M26317 might be a potential biomarker that correlate with Lauren’s classification, lymph node and distant metastasis (Li et al., 2018). The lncRNAs RP11-119F7.4, C5orf66-AS1 and DLEU2 were differentially expressed in GC tissue and non-tumour gastric tissue, and were predominantly correlated with Lauren histologic classification of GC (Sun et al., 2015; Zhou Q. et al., 2020; Hu et al., 2022). In addition to this, some lncRNAs expressed in some specific tissue (i.e. GAS5 and H19 expressed in embryo tissue) that can be targeted using nucleic acid-based drugs, small molecule inhibitors, and gene-editing methods at different functional levels to provide various therapeutic options (Arun G. et al., 2018).

In clinical practice, the lncRNAs expression might be tested in patients’ fluid samples from whole blood, serum or plasma, and tissue samples from gastric carcinoma tumour tissue and surrounding tissues or adjacent non-cancer tissues using qRT-PCT technique, to diagnose and predict the lymphatic metastasis and distal metastasis of GC. The single lncRNAs or combined lncRNAs or combined lncRNAs with the well-established biomarkers (CA12-5, CA72-4 and CA19-9) are promising biomarkers for assessing the diagnosis and prognosis of advanced gastric carcinoma. According to present studies, lncRNAs has potential valuable of being the biomarker for patients with GC in clinical settings. Therefore, the effects of lncRNAs on diagnosis and prognosis of GC are summarized in Table 4.

**TABLE 4:** The correlation between LncRNAs and diagnosis or prognosis in metastatic GC.

LncRNA	Expression in GC	Type of clinical sample	Potential roles of detecting the expression of LncRNAs for GC diagnosis and prognosis	References
AOC4P	Up	Tumour tissue	Correlates with poor overall and disease-free survival, expression was correlated with lymph vascular invasion	Zhang et al. (2019b)
AFAP1--AS1	Up	Tumour tissue	Correlates with the poor survival rates of GC patients, increased in the primary tumour tissues of GC patients with lymph node metastasis or tumour node metastasis stage	Zhao et al. (2018)
B3GALT5-AS1	Up	Serum	Correlates with tumour Node Metastasis (TNM) stage, and lymph node metastasis	Feng et al. (2020)
CASC15	Up	Tumour tissue	Correlates with a poor prognosis for patients suffering from GC	Wu et al. (2018)
CCAT2	Up	Tumour tissue	Correlates with tumour size, lymph node metastasis and TNM stage in GC patients	Wang et al. (2016c)
CTSLP4	Down	Tumour tissue	Correlates with tumour local invasion, TNM stage, lymph node metastasis, and prognosis of GC patients	Pan et al. (2021)
DANCR	Up	Tumour tissues, serum	Correlates with tumour size, TNM stage, lymphatic metastasis, and invasion depth	Pan et al. (2018)
DLGAP1-AS2	Up	Tumour tissue	Correlates with age, lymphatic, and vascular invasion in internal samples	Soltani et al. (2022)
DLX6-AS1	Up	Tumour tissue	Correlates with advanced clinical stage, lymph node metastasis and distant metastasis, decreased survival	Fu et al. (2019)
DLX6-AS1	Up	Tumour tissue	Correlates with T3/T4 invasion, distant metastasis, and poor clinical prognosis	Yu et al. (2020)
H19	Up	Serum	H19 levels is significantly decreased after compared with before surgery in patients with GC	Zhou et al. (2020b)
HNF1A-AS1	Up	Tumour tissue	A potential therapeutic target for alleviating GC chemoresistance	Jiang et al. (2022)
HOTAIR	Up	Tumour tissue	Correlates with poor prognosis in GC patients	Dong et al. (2019)
HOTAIR	Up	Tumour tissue	Correlates with lymph node metastasis and TNM stage,, was a predictor of poor over-all survival in GC patients	Xu et al. (2013)
HOTTIP	Up	Serum	Correlates with invasion depth and TNM stage	Rui et al. (2018)
HULC	Up	Tumour tissue	Correlates with lymph node metastasis, distant metastasis, and advanced tumours node metastasis stages	Zhao et al. (2014)
HULC	Up	Serum	Correlated with tumour size, lymph node metastasis, distant metastasis, tumour-node-metastasis stage, and *H. pylori* infection	Jin et al. (2016)
LINC00261	Down	Tumour tissue	Correlates with advanced tumour status and clinical stage as well as poor prognostic outcome	Yu et al. (2017)
LINC00978	Up	Tumour tissues, serum	Correlates with tumour size, lymphatic metastasis and TNM stage	Fu et al. (2018)
LINC00184	Up	Tumour tissue	Correlates with a worse prognosis	Piao et al. (2021)
LINC01061	Up	Tumour tissues、serum	Correlates with the clinicopathological features and survival time	Liang et al. (2021)
LINC01094	Up	Tumour tissue	Correlates with poor overall survival	Ye et al. (2022)
LINC01272	Up	Tumour tissue	Correlates with advanced GC staging and lymph node metastasis	Leng et al. (2020)
LINC01503	Up	Tumour tissue	Correlates with lymph node metastasis, TNM stage, and poor prognosis of GCA patients	Guo et al. (2021)
lincRNA-p21	Down	Tumour tissue	Correlates with higher invasion depth grade, more distant metastasis and advanced TNM stage	Chen et al. (2017)
Lnc01614	Up	Tumour tissue	Correlates with higher tumours staging, greater lymph node metastasis and distant metastasis rates, and lower overall survival rate	Dong et al. (2018)
Loc490	Down	Tumour tissue	Correlates with lymph node metastasis negatively and vein/nerve invasion, while it correlated positively with overall and disease-free survival	He et al. (2020)
MALAT1	Up	Tumour tissue	The expression of MALAT1 was significantly elevated in various GC cell lines and GC tumour tissues compared to normal cell lines and tumour tissues	Lee et al. (2017)
MALAT1	Up	Tumour tissue	Correlated with local invasion, lymph node metastasis, TNM stage, shorter survival, and poor prognosis	Li et al. (2017)
NR027113	Up	Tumour tissue	Positively correlates with lymph node metastasis and distant metastasis	Chen et al. (2019)
p4516	Up	Tumour tissue	Correlates with worse clinical outcomes	Nie et al. (2019)
PCAT6	Up	Tumour tissue	Negatively correlated to prognosis, tumour size, TNM stage and metastasis of GC	Xu et al. (2018c)
PCED1B-AS1	Up	Tumour tissue	Correlates with tumour size, TNM stage and lymph node metastasis in GC patients	Ren et al. (2021)
RP11-731F5.2	Up	Serum	The serum levels of RP11-731F5.2 in GC patients were significantly higher than those in healthy controls, correlates with vival time	Jing et al. (2020)
SNHG1	Up	Tumour tissue	Correlates with poor prognosis	Guo et al. (2019a)
SNHG6	Up	Tumour tissue	Correlates with invasion depth, lymph node metastasis, distant metastasis, and TNM stage and predicted poor prognosis	Yan et al. (2017)
SNHG7	Up	Tumour tissue	Positively correlated with TNM stage, depth of invasion, lymph-node metastasis, distant metastasis and an independent poor prognostic factor for overall survival in GC patients	Zhang et al. (2020b)
SPRY4-IT1	Down	Tumour tissue	Associates with larger tumour size, advanced pathological stage, deeper depth of invasion and lymphatic metastasis	Xie et al. (2015)
TMPO-AS1	Up	Tumour tissue	Correlates with aggressive clinicopathologic characteristics and poor overall survival	Sun and Han, (2020)
TP73-AS1	Up	Tumour tissue	Associated with tumour size, TNM stage, and overall survival	Wei et al. (2018)
TTTY15	Up	Tumour tissue	Associates with advanced TNM stage and poor tumour differentiation	Zheng et al. (2022)
UBE2CP3	Up	Tumour tissue	Associates with poor prognosis in GC	Li et al. (2021)
VIM-AS1	Up	Tumour tissue	Relates to the prognosis of patients with GC	Sun et al. (2020)
XLOC_006753	Up	Tumour tissue	Correlates with tumour progression (MDR reversal)	Zeng et al. (2018)
ZEB2-AS1	Up	Tumour tissue	Correlates with tumour progression	Wang et al. (2019a)
ZFAS1	Up	Tumour tissues, serum, serum exosomes	Correlated with lymphatic metastasis and TNM stage	Lei et al. (2017)
ZFAS1	Up	Tumour tissues, plasmas	Correlates with tumour progression	Hu et al. (2016)

## Perspectives

LncRNAs play an important role in the development of GC. Some identified oncogenic lncRNAs overexpressed in gastric cancerous tissue, such as H19, HOTAIR, and MALAT1, whereas others are expressed in the tissue from gastric carcinoma at a low level, such as LincRNA-p21, LINC00261, CTLSP4 and SPRY4-IT1. LncRNAs regulate EMT process by targeting EMT-related signalling pathways directly (i.e., H19, HOTAIR, ZEB2-AS1, LincRNA-p21 and SNHG1), or function as ceRNAs (i.e., H19, HOTTIP, MALAT1, SNHG1 and SNHG6) for tumour suppressive miRNAs. Furthermore, dysfunction of lncRNAs regulates apoptosis and cell cycle in GC cell lines, for example, SNHG6 function as a positive regulator for BCL-2 gene expression. In addition, SNHG6 implicated in initiation and EMT-induced metastasis of GC by regulating ZEB1 expression. Dysregulated lncRNAs (SNGH6, HOTTIP, H19, HNF1A-AS1 and ZFAS1) exert the functional role in chemoresistance leading to enhanced EMT ability in GC cell lines and tissues. Aberrant expression of H19 is involved in progression and metastasis in numerous cancer through regulating various of targeted genes. For example, it regulates VEGF expression by competitively binding miR-138 in glioma (Liu Z. Z. et al., 2020). H19/miRNA-140 axis promotes ovarian cancer cell migration by upregulating Wnt1 expression (Wang and Gao, 2021). Additionally, H19 upregulates PFTK1 expression through targeting miR-194 in pancreatic cancer cells (Sun Y. et al., 2019). Since targeting lncRNAs are currently under development by researchers, H19 might be a promising target in the treatment of patients with advanced GC.

In addition to being a potential therapeutic target for GC, another important clinical value of lncRNAs is as a diagnostic marker (differentially expression in GC and surrounding tissues or in GC patients and health individuals) or prognostic markers (association with Lauren’s classification, TNM stage, lymph node metastasis and overall survival time). It is likely that the overexpression of serum-derived lncRNAs, such as H19, HOTTIP, DANCR and HULC, may be an early event in tumorigenesis of the GC. The upregulation of tumour tissue-derived lncRNAs (HOTAIR, MALAT1, SHNG1 and SHNG6) might be adverse prognostic factors of GC. To ensure high specificity and sensitivity of the diagnosis and prognosis of GC, the expression level of lncRNAs, both diagnostic and prognostic markers, can be used alone or in combination with existing markers. The above insights may help to provide strategies for basic research and clinical diagnosis and treatment with lncRNAs. However, more in-depth investigations are still required to verify the practicality of lncRNAs in clinical application.

## References

[B1] Albrecht L. V. Tejeda-Muñoz N. Cell E. M. D. R. J. A. R. O. Biology D. (2021). Cell Biology of canonical Wnt signaling. Annu. Rev. Cell Dev. Biol. 37, 369–389. 10.1146/annurev-cellbio-120319-023657 34196570

[B2] Amin M. B. Greene F. L. Edge S. B. Compton C. C. Gershenwald J. E. Brookland R. K. (2017). The Eighth Edition AJCC Cancer Staging Manual: Continuing to build a bridge from a population-based to a more "personalized" approach to cancer staging. Ca. Cancer J. Clin. 67, 93–99. 10.3322/caac.21388 28094848

[B3] Arita T. Ichikawa D. Konishi H. Komatsu S. Shiozaki A. Shoda K. (2013). Circulating long non-coding RNAs in plasma of patients with gastric cancer. Anticancer Res. 33, 3185–3193. 23898077

[B4] Arun G. Diermeier S. D. Spector D. L. (2018a). Therapeutic targeting of long non-coding RNAs in cancer. Trends Mol. Med. 24, 257–277. 10.1016/j.molmed.2018.01.001 29449148 PMC5840027

[B5] Arun K. Arunkumar G. Bennet D. Chandramohan S. M. Murugan A. K. Munirajan A. K. (2018b). Comprehensive analysis of aberrantly expressed lncRNAs and construction of ceRNA network in gastric cancer. Oncotarget 9, 18386–18399. 10.18632/oncotarget.24841 29719612 PMC5915079

[B6] Ba M. C. Ba Z. Long H. Cui S. Z. Gong Y. F. Yan Z. F. (2020). LncRNA AC093818.1 accelerates gastric cancer metastasis by epigenetically promoting PDK1 expression. Cell Death Dis. 11, 64. 10.1038/s41419-020-2245-2 31988283 PMC6985138

[B7] Barriere G. Fici P. Gallerani G. Fabbri F. Rigaud M. (2015). Epithelial mesenchymal transition: A double-edged sword. Clin. Transl. Med. 4, 14. 10.1186/s40169-015-0055-4 25932287 PMC4409604

[B8] Beckedorff F. C. Amaral M. S. Deocesano-Pereira C. Verjovski-Almeida S. (2013). Long non-coding RNAs and their implications in cancer epigenetics. Biosci. Rep. 33, e00061. 10.1042/BSR20130054 23875687 PMC3759304

[B9] Bhagwat A. S. Vakoc C. R. (2015). Targeting transcription factors in cancer. Trends Cancer 1, 53–65. 10.1016/j.trecan.2015.07.001 26645049 PMC4669894

[B10] Bhan A. Soleimani M. Mandal S. S. (2017). Long noncoding RNA and cancer: A new paradigm. Cancer Res. 77, 3965–3981. 10.1158/0008-5472.CAN-16-2634 28701486 PMC8330958

[B11] Blum M. A. Takashi T. Suzuki A. Ajani J. A. (2013). Management of localized gastric cancer. J. Surg. Oncol. 107, 265–270. 10.1002/jso.23183 23303654

[B12] Böhmdorfer G. Wierzbicki A. T. (2015). Control of chromatin structure by long noncoding RNA. Trends Cell Biol. 25, 623–632. 10.1016/j.tcb.2015.07.002 26410408 PMC4584417

[B13] Bürglin T. R. Affolter M. (2016). Homeodomain proteins: An update. Chromosoma 125, 497–521. 10.1007/s00412-015-0543-8 26464018 PMC4901127

[B14] Canzio D. Nwakeze C. L. Horta A. Rajkumar S. M. Coffey E. L. Duffy E. E. (2019). Antisense lncRNA transcription mediates DNA demethylation to drive stochastic protocadherin α promoter choice. Cell 177, 639–653. e15. 10.1016/j.cell.2019.03.008 30955885 PMC6823843

[B15] Chan J. J. Tay Y. (2018). Noncoding RNA:RNA regulatory networks in cancer. Int. J. Mol. Sci. 19, 1310. 10.3390/ijms19051310 PMC5983611 29702599

[B16] Chan S. H. Wang L. H. (2015). Regulation of cancer metastasis by microRNAs. J. Biomed. Sci. 22, 9. 10.1186/s12929-015-0113-7 25614041 PMC4318216

[B17] Chao Z. Na A. Chunyan C. Guodong W. (2020). lncRNA HOXC-AS1 promotes gastric cancer via binding eIF4AIII by activating Wnt/β-catenin signaling. J. Gene Med. 22, e3202. 10.1002/jgm.3202 32307743

[B18] Chava S. Gayatri M. B. Reddy A. B. M. (2019). “Chapter 3 - EMT contributes to chemoresistance in pancreatic cancer,” in Breaking tolerance to pancreatic cancer unresponsiveness to chemotherapy. Editor NAGARAJU G. P. (Massachusetts, US: Academic Press).

[B19] Chen N. Zhu X. Zhu Y. Shi J. Zhang J. Tang C. (2021a). The regulatory relationship and function of LncRNA FAM225A-miR-206-ADAM12 in gastric cancer. Am. J. Transl. Res. 13, 8632–8652. 34539984 PMC8430187

[B20] Chen Q. F. Hu C. F. Sun K. Lang Y. P. (2019). LncRNA NR027113 promotes malignant progression of gastric carcinoma via EMT signaling pathway. Eur. Rev. Med. Pharmacol. Sci. 23, 4746–4755. 10.26355/eurrev_201906_18056 31210301

[B21] Chen X. Chen Z. Yu S. Nie F. Yan S. Ma P. (2018). Long noncoding RNA LINC01234 functions as a competing endogenous RNA to regulate CBFB expression by sponging miR-204-5p in gastric cancer. Clin. Cancer Res. 24, 2002–2014. 10.1158/1078-0432.CCR-17-2376 29386218

[B22] Chen Y. Wei G. Xia H. Yu H. Tang Q. Bi F. (2017). Down regulation of lincRNA-p21 contributes to gastric cancer development through Hippo-independent activation of YAP. Oncotarget 8, 63813–63824. 10.18632/oncotarget.19130 28969031 PMC5609963

[B23] Chen Y. Zhang R. (2021). Long non-coding RNA AL139002.1 promotes gastric cancer development by sponging microRNA-490-3p to regulate Hepatitis A Virus Cellular Receptor 1 expression. Bioengineered 12, 1927–1938. 10.1080/21655979.2021.1922329 34002670 PMC8806325

[B24] Chen Z. Li Y. Tan B. Li F. Zhao Q. Fan L. (2021b). Long non-coding RNA ASNR targeting miR-519e-5p promotes gastric cancer development by regulating FGFR2. Front. Cell Dev. Biol. 9, 679176. 10.3389/fcell.2021.679176 34307360 PMC8299726

[B25] Choi S. W. Kim H. W. Nam J. W. (2019). The small peptide world in long noncoding RNAs. Brief. Bioinform. 20, 1853–1864. 10.1093/bib/bby055 30010717 PMC6917221

[B26] Cristescu R. Lee J. Nebozhyn M. Kim K.-M. Ting J. C. Wong S. S. (2015). Molecular analysis of gastric cancer identifies subtypes associated with distinct clinical outcomes. Nat. Med. 21, 449–456. 10.1038/nm.3850 25894828

[B27] Cui G. Cai F. Ding Z. Pathology L. G. J. H. (2019). HMGB2 promotes the malignancy of human gastric cancer and indicates poor survival outcome. Hum. Pathol. 84, 133–141. 10.1016/j.humpath.2018.09.017 30296520

[B28] D'Elia L. Rossi G. Ippolito R. Cappuccio F. P. Strazzullo P. (2012). Habitual salt intake and risk of gastric cancer: A meta-analysis of prospective studies. Clin. Nutr. 31, 489–498. 10.1016/j.clnu.2012.01.003 22296873

[B29] Dai J. Nishi A. Li Z. X. Zhang Y. Zhou T. You W. C. (2021a). DNA methylation signatures associated with prognosis of gastric cancer. BMC Cancer 21, 610. 10.1186/s12885-021-08389-0 34034702 PMC8152126

[B30] Dai W. Xiao Y. Tang W. Li J. Hong L. Zhang J. (2021b). Identification of an EMT-related gene signature for predicting overall survival in gastric cancer. Front. Genet. 12, 661306. 10.3389/fgene.2021.661306 34249086 PMC8264558

[B31] Dai X. Ren T. Zhang Y. Nan N. (2021c). Methylation multiplicity and its clinical values in cancer. Expert Rev. Mol. Med. 23, e2. 10.1017/erm.2021.4 33787478 PMC8086398

[B32] Deshmukh A. P. Vasaikar S. V. Tomczak K. Tripathi S. Den Hollander P. Arslan E. (2021). Identification of EMT signaling cross-talk and gene regulatory networks by single-cell RNA sequencing. Proc. Natl. Acad. Sci. U. S. A. 118, e2102050118. 10.1073/pnas.2102050118 33941680 PMC8126782

[B33] Digklia A. Wagner A. D. (2016). Advanced gastric cancer: Current treatment landscape and future perspectives. World J. Gastroenterol. 22, 2403–2414. 10.3748/wjg.v22.i8.2403 26937129 PMC4768187

[B34] Dong X. He X. Guan A. Huang W. Jia H. Huang Y. (2019). Long non-coding RNA Hotair promotes gastric cancer progression via miR-217-GPC5 axis. Life Sci. 217, 271–282. 10.1016/j.lfs.2018.12.024 30557546

[B35] Dong Y. Wang Z. G. Chi T. S. (2018). Long noncoding RNA Lnc01614 promotes the occurrence and development of gastric cancer by activating EMT pathway. Eur. Rev. Med. Pharmacol. Sci. 22, 1307–1314. 10.26355/eurrev_201803_14472 29565488

[B36] Du Z. Sun T. Hacisuleyman E. Fei T. Wang X. Brown M. (2016). Integrative analyses reveal a long noncoding RNA-mediated sponge regulatory network in prostate cancer. Nat. Commun. 7, 10982. 10.1038/ncomms10982 26975529 PMC4796315

[B231] Dykes I. M. Emanueli C. (2017). Transcriptional and Post-transcriptional Gene Regulation by Long Non-coding RNA. Genomics, Proteomics and Bioinformatics 15, 177–186. 10.1016/j.gpb.2016.12.005 PMC5487525 28529100

[B37] Fatima R. Akhade V. S. Pal D. Rao S. M. (2015). Long noncoding RNAs in development and cancer: Potential biomarkers and therapeutic targets. Mol. Cell. Ther. 3, 5. 10.1186/s40591-015-0042-6 26082843 PMC4469312

[B38] Feng F. Tian Y. Xu G. Liu Z. Liu S. Zheng G. (2017). Diagnostic and prognostic value of CEA, CA19-9, AFP and CA125 for early gastric cancer. BMC Cancer 17, 737. 10.1186/s12885-017-3738-y 29121872 PMC5679342

[B39] Feng W. Zong W. Li Y. Shen X. Cui X. Ju S. (2020). Abnormally expressed long noncoding RNA B3GALT5-AS1 may serve as a biomarker for the diagnostic and prognostic of gastric cancer. J. Cell. Biochem. 121, 557–565. 10.1002/jcb.29296 31338903

[B40] Fu M. Huang Z. Zang X. Pan L. Liang W. Chen J. (2018). Long noncoding RNA LINC00978 promotes cancer growth and acts as a diagnostic biomarker in gastric cancer. Cell Prolif. 51. 10.1111/cpr.12425 PMC6528885 29271006

[B41] Fu X. Tian Y. Kuang W. Wen S. Guo W. (2019). Long non-coding RNA DLX6-AS1 silencing inhibits malignant phenotypes of gastric cancer cells. Exp. Ther. Med. 17, 4715–4722. 10.3892/etm.2019.7521 31105791 PMC6507526

[B42] Gaianigo N. Melisi D. Carbone C. (2017). EMT and treatment resistance in pancreatic cancer. Cancers 9, 122. 10.3390/cancers9090122 PMC5615337 28895920

[B43] Gao M. Liu L. Yang Y. Li M. Ma Q. Chang Z. (2021). LncRNA HCP5 induces gastric cancer cell proliferation, invasion, and EMT processes through the miR-186-5p/wnt5a Axis under hypoxia. Front. Cell Dev. Biol. 9, 663654. 10.3389/fcell.2021.663654 34178988 PMC8226141

[B44] Gharaibeh L. Elmadany N. Alwosaibai K. Alshaer W. (2020). Notch1 in cancer therapy: Possible clinical implications and challenges. Mol. Pharmacol. 98, 559–576. 10.1124/molpharm.120.000006 32913140

[B45] Gong P. Qiao F. Wu H. Cui H. Li Y. Zheng Y. (2018). LncRNA UCA1 promotes tumor metastasis by inducing miR-203/ZEB2 axis in gastric cancer. Cell Death Dis. 9, 1158. 10.1038/s41419-018-1170-0 30464170 PMC6249325

[B46] Grumati P. Dikic I. (2018). Ubiquitin signaling and autophagy. J. Biol. Chem. 293, 5404–5413. 10.1074/jbc.TM117.000117 29187595 PMC5900779

[B47] Guo L. Ma H. Kong Y. Leng G. Liu G. Zhang Y. (2022). Long non-coding RNA TNK2 AS1/microRNA-125a-5p axis promotes tumor growth and modulated phosphatidylinositol 3 kinase/AKT pathway. J. Gastroenterol. Hepatol. 37, 124–133. 10.1111/jgh.15683 34494305

[B48] Guo W. Huang J. Lei P. Guo L. Li X. (2019a). LncRNA SNHG1 promoted HGC-27 cell growth and migration via the miR-140/ADAM10 axis. Int. J. Biol. Macromol. 122, 817–823. 10.1016/j.ijbiomac.2018.10.214 30391432

[B49] Guo W. Liang X. Liu L. Guo Y. Shen S. Liang J. (2019b). MiR-6872 host gene SEMA3B and its antisense lncRNA SEMA3B-AS1 function synergistically to suppress gastric cardia adenocarcinoma progression. Gastric Cancer 22, 705–722. 10.1007/s10120-019-00924-0 30656427

[B50] Guo Y. Sun P. Guo W. Dong Z. (2021). Long non-coding RNA LINC01503 promotes gastric cardia adenocarcinoma progression via miR-133a-5p/VIM Axis and EMT process. Dig. Dis. Sci. 66, 3391–3403. 10.1007/s10620-020-06690-9 33200343

[B51] He C. Qi W. Wang Z. (2021a). Effect and mechanism of downregulating the long-chain noncoding RNA TM4SF1-AS1 on the proliferation, apoptosis and invasion of gastric cancer cells. World J. Surg. Oncol. 19, 226. 10.1186/s12957-021-02334-y 34330293 PMC8325262

[B52] He S. Zhang H. Xiao Z. Bhushan S. Gao K. Bioengineered W. W. J. (2021b). The interaction of TEA domain transcription factor 4 (TEAD4) and Yes-associated protein 1 (YAP1) promoted the malignant process mediated by serum/glucocorticoid regulated kinase 1 (SGK1). Bioengineered 12, 601–614. 10.1080/21655979.2021.1882142 33517828 PMC8806348

[B53] He Z. Duan Z. Chen L. Li B. Zhou Y. (2020). Long non-coding RNA Loc490 inhibits gastric cancer cell proliferation and metastasis by upregulating RNA-binding protein Quaking. Aging 12, 17681–17693. 10.18632/aging.103876 32931453 PMC7521539

[B54] Howlader N. Noone A. M. Krapcho M. Miller D. Brest A. Yu M. (2020). SEER cancer statistics review, 1975-2017. Bethesda, MD: National Cancer Institute.

[B55] Hu J. Wang M. Yang Y. Xing Y. Li S. (2022). LncRNA DLEU2 silencing impedes the migration, invasion and EMT in gastric cancer cell by suppressing PI3K/AKT signaling pathway. Immunopharmacol. Immunotoxicol. 2022, 1–13. 10.1080/08923973.2022.2078727 35736813

[B56] Hu Z. Fubing W. Hao C. Qian T. Shili Q. Shanshan C. (2016). Increased expression of long-noncoding RNA ZFAS1 is associated with epithelial-mesenchymal transition of gastric cancer. Aging 8, 2023–2038. 10.18632/aging.101048 27654478 PMC5076450

[B57] Huang D. Zhang K. Zheng W. Zhang R. Chen J. Du N. (2021). Long noncoding RNA SGO1-AS1 inactivates TGFβ signaling by facilitating TGFB1/2 mRNA decay and inhibits gastric carcinoma metastasis. J. Exp. Clin. Cancer Res. 40, 342. 10.1186/s13046-021-02140-0 34706749 PMC8555099

[B58] Huang J. Q. Sridhar S. Chen Y. Hunt R. H. (1998). Meta-analysis of the relationship between *Helicobacter pylori* seropositivity and gastric cancer. Gastroenterology 114, 1169–1179. 10.1016/s0016-5085(98)70422-6 9609753

[B59] Huang L. Wu R.-L. Xu A. M. (2015). Epithelial-mesenchymal transition in gastric cancer. Am. J. Transl. Res. 7, 2141–2158. 26807164 PMC4697696

[B60] Huang Y. Zhang J. Hou L. Wang G. Liu H. Zhang R. (2017). LncRNA AK023391 promotes tumorigenesis and invasion of gastric cancer through activation of the PI3K/Akt signaling pathway. J. Exp. Clin. Cancer Res. 36, 194. 10.1186/s13046-017-0666-2 29282102 PMC5745957

[B61] Jafarzadeh M. Soltani B. M. (2020). Long noncoding RNA LOC400043 (LINC02381) inhibits gastric cancer progression through regulating Wnt signaling pathway. Front. Oncol. 10, 562253. 10.3389/fonc.2020.562253 33194632 PMC7645048

[B62] Ji Z. Tang T. Chen M. Dong B. Sun W. Wu N. (2021). C-Myc-activated long non-coding RNA LINC01050 promotes gastric cancer growth and metastasis by sponging miR-7161-3p to regulate SPZ1 expression. J. Exp. Clin. Cancer Res. 40, 351. 10.1186/s13046-021-02155-7 34749766 PMC8573944

[B63] Jiang L. Zhang Y. Su P. Ma Z. Ye X. Kang W. (2022). Long non-coding RNA HNF1A-AS1 induces 5-FU resistance of gastric cancer through miR-30b-5p/EIF5A2 pathway. Transl. Oncol. 18, 101351. 10.1016/j.tranon.2022.101351 35092904 PMC8802127

[B64] Jiang X. Ding W. Shen W. Jin J. (2020). H19/miR-152-3p/TCF4 axis increases chemosensitivity of gastric cancer cells through suppression of epithelial-mesenchymal transition. Transl. Cancer Res. 9, 3915–3925. 10.21037/tcr-20-1736 35117758 PMC8798114

[B65] Jin C. Shi W. Wang F. Shen X. Qi J. Cong H. (2016). Long non-coding RNA HULC as a novel serum biomarker for diagnosis and prognosis prediction of gastric cancer. Oncotarget 7, 51763–51772. 10.18632/oncotarget.10107 27322075 PMC5239513

[B66] Jing R. Liu S. Jiang Y. Zong W. Ju S. Cui M. (2020). Determination of serum RP11-731F5.2 as a noninvasive biomarker for gastric cancer diagnosis and prognosis. Pathol. Res. Pract. 216, 153261. 10.1016/j.prp.2020.153261 33176259

[B67] Kalluri R. Weinberg R. A. (2009). The basics of epithelial-mesenchymal transition. J. Clin. Invest. 119, 1420–1428. 10.1172/JCI39104 19487818 PMC2689101

[B68] Kapranov P. Cheng J. Dike S. Nix D. A. Duttagupta R. Willingham A. T. (2007). RNA maps reveal new RNA classes and a possible function for pervasive transcription. Science 316, 1484–1488. 10.1126/science.1138341 17510325

[B69] Karagkouni D. Karavangeli A. Paraskevopoulou M. D. Biology A. G. H. J. M. I. M. (2021). Characterizing miRNA-lncRNA interplay. Methods Mol. Biol. 2372, 243–262. 10.1007/978-1-0716-1697-0_21 34417757

[B70] Kim D. H. Xing T. Yang Z. Dudek R. Lu Q. Chen Y. H. (2017). Epithelial mesenchymal transition in embryonic development, tissue repair and cancer: A comprehensive overview. J. Clin. Med. 7, E1. 10.3390/jcm7010001 29271928 PMC5791009

[B71] Kong L. Sun Y. Chen M. Dai Y. Experimental Z. L. J. Medicine T. (2020). Downregulation of microRNA-320a inhibits proliferation and induces apoptosis of retinoblastoma cells via targeting TUSC3. Exp. Ther. Med. 20, 9. 10.3892/etm.2020.9137 PMC7471862 32934674

[B72] Kontomanolis E. N. Koutras A. Syllaios A. Schizas D. Mastoraki A. Garmpis N. (2020). Role of oncogenes and tumor-suppressor genes in carcinogenesis: A review. Anticancer Res. 40, 6009–6015. 10.21873/anticanres.14622 33109539

[B73] Kyrgiou M. Kalliala I. Markozannes G. Gunter M. J. Paraskevaidis E. Gabra H. (2017). Adiposity and cancer at major anatomical sites: Umbrella review of the literature. Bmj 356, j477. 10.1136/bmj.j477 28246088 PMC5421437

[B74] Ladeiras-Lopes R. Pereira A. K. Nogueira A. Pinheiro-Torres T. Pinto I. Santos-Pereira R. (2008). Smoking and gastric cancer: Systematic review and meta-analysis of cohort studies. Cancer Causes Control 19, 689–701. 10.1007/s10552-008-9132-y 18293090

[B75] Lamouille S. Xu J. Derynck R. (2014). Molecular mechanisms of epithelial–mesenchymal transition. Nat. Rev. Mol. Cell Biol. 15, 178–196. 10.1038/nrm3758 24556840 PMC4240281

[B76] Latchman D. S. (1993). Transcription factors: An overview. Int. J. Exp. Pathol. 74, 417–422. 8217775 PMC2002184

[B77] Lauren P. (1965). The two histological main types of gastric carcinoma: Diffuse and SO-called intestinal-type carcinoma. AN attempt at a histo-clinical classification. Acta Pathol. Microbiol. Scand. 64, 31–49. 10.1111/apm.1965.64.1.31 14320675

[B78] Lee N. K. Lee J. H. Ivan C. Ling H. Zhang X. Park C. H. (2017). MALAT1 promoted invasiveness of gastric adenocarcinoma. BMC Cancer 17, 46. 10.1186/s12885-016-2988-4 28077118 PMC5225525

[B79] Lee S. K. Lee J. H. Lee N. K. Park C. H. (2015). Sa1983 long non-coding RNA MALAT-1 promote invasion and migration of gastric cancer. Gastroenterology 148, S374–S374. 10.1016/s0016-5085(15)31256-7

[B80] Lei P. Wei L. Min F. Zhen-Hua H. Xia L. Wen Z. (2017). Exosomes-mediated transfer of long noncoding RNA ZFAS1 promotes gastric cancer progression. J. Cancer Res. Clin. Oncol. 143, 991–1004. 10.1007/s00432-017-2361-2 28285404 PMC11819301

[B81] Leng X. Liu G. Wang S. Song J. Zhang W. Zhang X. (2020). LINC01272 promotes migration and invasion of gastric cancer cells via EMT. Onco. Targets. Ther. 13, 3401–3410. 10.2147/OTT.S242073 32368096 PMC7184168

[B82] Li D. She J. Hu X. Zhang M. Sun R. Qin S. (2021). The ELF3-regulated lncRNA UBE2CP3 is over-stabilized by RNA–RNA interactions and drives gastric cancer metastasis via miR-138-5p/ITGA2 axis. Oncogene 40, 5403–5415. 10.1038/s41388-021-01948-6 34274947 PMC8413130

[B83] Li D. Wang J. Zhang M. Hu X. She J. Qiu X. (2020a). LncRNA MAGI2-AS3 is regulated by BRD4 and promotes gastric cancer progression via maintaining ZEB1 overexpression by sponging miR-141/200a. Mol. Ther. Nucleic Acids 19, 109–123. 10.1016/j.omtn.2019.11.003 31837602 PMC6920306

[B84] Li D. Xu M. Wang Z. Huang P. Huang C. Chen Z. (2022). The EMT-induced lncRNA NR2F1-AS1 positively modulates NR2F1 expression and drives gastric cancer via miR-29a-3p/VAMP7 axis. Cell Death Dis. 13, 84. 10.1038/s41419-022-04540-2 35082283 PMC8791943

[B85] Li H. Chen X. Gao Y. Wu J. Zeng F. Song F. (2015). XBP1 induces snail expression to promote epithelial- to-mesenchymal transition and invasion of breast cancer cells. Cell. Signal. 27, 82–89. 10.1016/j.cellsig.2014.09.018 25280941

[B86] Li J. Gao J. Tian W. Li Y. Zhang J. (2017). Long non-coding RNA MALAT1 drives gastric cancer progression by regulating HMGB2 modulating the miR-1297. Cancer Cell Int. 17, 44. 10.1186/s12935-017-0408-8 28396617 PMC5383984

[B87] Li L. Wang Y. Y. Mou X. Z. Ye Z. Y. Zhao Z. S. (2018). Up-regulation of long noncoding RNA M26317 correlates with tumor progression and poor prognosis in gastric cancer. Hum. Pathol. 78, 44–53. 10.1016/j.humpath.2018.04.008 29698700

[B88] Li N. Ouyang Y. Chen S. Peng C. He C. Hong J. (2020b). Integrative analysis of differential lncRNA/mRNA expression profiling in *Helicobacter pylori* infection-associated gastric carcinogenesis. Front. Microbiol. 11, 880. 10.3389/fmicb.2020.00880 32457731 PMC7225608

[B89] Li X. M. Jiao Y. Y. Luan B. H. Wu H. X. Wang R. R. Zhong J. (2020c). Long non-coding RNA MIAT promotes gastric cancer proliferation and metastasis via modulating the miR-331-3p/RAB5B pathway. Oncol. Lett. 20, 355. 10.3892/ol.2020.12219 33154765 PMC7608069

[B90] Li Y. Jiang L. Lv S. Xu H. Fan Z. He Y. (2019). E2F6-mediated lncRNA CASC2 down-regulation predicts poor prognosis and promotes progression in gastric carcinoma. Life Sci. 232, 116649. 10.1016/j.lfs.2019.116649 31301415

[B91] Li Y. Yan J. Wang Y. Wang C. Zhang C. Li G. (2020d). LINC00240 promotes gastric cancer cell proliferation, migration and EMT via the miR-124-3p/DNMT3B axis. Cell biochem. Funct. 38, 1079–1088. 10.1002/cbf.3551 32526811

[B92] Li Y. Zhao Z. Xu C. Zhou Z. Zhu Z. You T. (2014). HMGA2 induces transcription factor Slug expression to promote epithelial-to-mesenchymal transition and contributes to colon cancer progression. Cancer Lett. 355, 130–140. 10.1016/j.canlet.2014.09.007 25218351

[B93] Liang M. Yunshan J. Ning W. (2022). Long non-coding RNA CCL2 promoted gastric cancer function via miR-128/PARP2 signal pathway. Bioengineered 13, 1602–1611. 10.1080/21655979.2021.2020548 35000531 PMC8805977

[B94] Liang W. Xia B. Yan M. Zhai G. Li M. (2021). Enhanced LINC01061 levels as a serum biomarker in gastric cancer and promotion of malignant transformation. Oncol. Res. Treat. 44, 242–251. 10.1159/000508310 33910210

[B95] Liang Y. Zhang C. D. Zhang C. Dai D. Q. (2020). DLX6-AS1/miR-204-5p/OCT1 positive feedback loop promotes tumor progression and epithelial-mesenchymal transition in gastric cancer. Gastric Cancer 23, 212–227. 10.1007/s10120-019-01002-1 31463827

[B96] Liao Z. Nie H. Wang Y. Luo J. Zhou J. Ou C. (2021). The emerging landscape of long non-coding RNAs in colorectal cancer metastasis. Front. Oncol. 11, 641343. 10.3389/fonc.2021.641343 33718238 PMC7947863

[B97] Lin H. Wang J. Wang T. Wu J. Wang P. Huo X. (2021). The LncRNA mir503hg/miR-224-5p/TUSC3 signaling cascade suppresses gastric cancer development via modulating ATF6 branch of unfolded protein response. Front. Oncol. 11, 708501. 10.3389/fonc.2021.708501 34381729 PMC8352579

[B98] Lin J. Song T. Li C. Mao W. (2020). GSK-3β in DNA repair, apoptosis, and resistance of chemotherapy, radiotherapy of cancer. Biochim. Biophys. Acta Mol. Cell Res. 1867, 118659. 10.1016/j.bbamcr.2020.118659 31978503

[B99] Liu C. Luo J. Zhao Y. T. Wang Z. Y. Zhou J. Huang S. (2018a). TWIST1 upregulates miR-214 to promote epithelial-to-mesenchymal transition and metastasis in lung adenocarcinoma. Int. J. Mol. Med. 42, 461–470. 10.3892/ijmm.2018.3630 29693173

[B100] Liu G. Xiang T. Wu Q. F. Wang W. X. (2016a). Long noncoding RNA H19-derived miR-675 enhances proliferation and invasion via RUNX1 in gastric cancer cells. Oncol. Res. 23, 99–107. 10.3727/096504015X14496932933575 26931432 PMC7838630

[B101] Liu H. Zhang Z. Wu N. Guo H. Zhang H. Fan D. (2018b). Integrative analysis of dysregulated lncRNA-associated ceRNA network reveals functional lncRNAs in gastric cancer. Genes (Basel) 9, 303. 10.3390/genes9060303 PMC6027299 29912172

[B102] Liu J. Liu L. Wan J. X. Song Y. (2017). Long noncoding RNA SNHG20 promotes gastric cancer progression by inhibiting p21 expression and regulating the GSK-3β/β-catenin signaling pathway. Oncotarget 8, 80700–80708. 10.18632/oncotarget.20959 29113337 PMC5655232

[B103] Liu J. Wang G. Zhao J. Liu X. Zhang K. Gong G. (2021). LncRNA H19 promoted the epithelial to mesenchymal transition and metastasis in gastric cancer via activating wnt/β-catenin signaling. Dig. Dis. 40, 436–447. 10.1159/000518627 34348271

[B104] Liu L. Zhao X. Zou H. Bai R. Yang K. Tian Z. (2016b). Hypoxia promotes gastric cancer malignancy partly through the HIF-1α dependent transcriptional activation of the long non-coding RNA GAPLINC. Front. Physiol. 7, 420. 10.3389/fphys.2016.00420 27729869 PMC5037220

[B105] Liu W. Zhang Z. Zhang Y. Chen X. Guo S. Lei Y. (2015a). THERAPYHMGB1-mediated autophagy modulates sensitivity of colorectal cancer cells to oxaliplatin via MEK/ERK signaling pathway. Cancer Biol. Ther. 16, 511–517. 10.1080/15384047.2015.1017691 25778491 PMC4622507

[B106] Liu Y. W. Sun M. Xia R. Zhang E. B. Liu X. H. Zhang Z. H. (2015b). LincHOTAIR epigenetically silences miR34a by binding to PRC2 to promote the epithelial-to-mesenchymal transition in human gastric cancer. Cell Death Dis. 6, e1802. 10.1038/cddis.2015.150 26136075 PMC4650715

[B107] Liu Z. Hu K. Wang X. Zhang Y. Wang W. Wu Y. (2022). LncRNA ACTA2-AS1 inhibits malignant phenotypes of gastric cancer cells. Open Med. 17, 266–279. 10.1515/med-2021-0406 PMC8854910 35274046

[B108] Liu Z. Q. He W. F. Wu Y. J. Zhao S. L. Wang L. Ouyang Y. Y. (2020a). LncRNA SNHG1 promotes EMT process in gastric cancer cells through regulation of the miR-15b/DCLK1/Notch1 axis. BMC Gastroenterol. 20, 156. 10.1186/s12876-020-01272-5 32423385 PMC7236477

[B109] Liu Z. Z. Tian Y. F. Wu H. Ouyang S. Y. Kuang W. L. (2020b). LncRNA H19 promotes glioma angiogenesis through miR-138/HIF-1α/VEGF axis. Neoplasma 67, 111–118. 10.4149/neo_2019_190121N61 31777264

[B110] Long Y. Wang X. Youmans D. T. Cech T. R. (2017). How do lncRNAs regulate transcription? Sci. Adv. 3, eaao2110. 10.1126/sciadv.aao2110 28959731 PMC5617379

[B111] Lu H. Zhang Q. Sun Y. Wu D. Liu L. (2020a). LINC00689 induces gastric cancer progression via modulating the miR-338-3p/HOXA3 axis. J. Gene Med. 22, e3275. 10.1002/jgm.3275 32926751

[B112] Lu J. Tang Y. Zhang W. Zhang S. Qi W. Ma G. (2020b). Expression and mechanism of long non-coding RNA DLGAP1-AS2 in gastric cancer. Tumor 40, 153–162.

[B113] Lu J. Xu Y. Xie W. Tang Y. Zhang H. Wang B. (2021). Long noncoding RNA DLGAP1-AS2 facilitates Wnt1 transcription through physically interacting with Six3 and drives the malignancy of gastric cancer. Cell Death Discov. 7, 255. 10.1038/s41420-021-00649-z 34545072 PMC8452735

[B114] Lv J. Zhang S. Liu Y. Li C. Guo T. Zhang S. (2021). NR2F1-AS1/miR-190a/PHLDB2 induces the epithelial-mesenchymal transformation process in gastric cancer by promoting phosphorylation of AKT3. Front. Cell Dev. Biol. 9, 688949. 10.3389/fcell.2021.688949 34746118 PMC8569557

[B115] Ma L. Bajic V. B. Zhang Z. (2013). On the classification of long non-coding RNAs. RNA Biol. 10, 925–933. 10.4161/rna.24604 23696037 PMC4111732

[B116] Ma L. Jiang Y. Wu N. (2022). Long non-coding RNA CCL2 promoted gastric cancer function via miR-128/PARP2 signal pathway. Bioengineered 13, 1602–1611. 10.1080/21655979.2021.2020548 35000531 PMC8805977

[B117] Ma P. Li L. Liu F. Zhao Q. (2020). HNF1A-Induced lncRNA HCG18 facilitates gastric cancer progression by upregulating DNAJB12 via miR-152-3p. Onco. Targets. Ther. 13, 7641–7652. 10.2147/OTT.S253391 32801777 PMC7413704

[B118] Macdonald B. T. Tamai K. Cell X. H. J. D. (2009). Wnt/beta-catenin signaling: Components, mechanisms, and diseases. Dev. Cell 17, 9–26. 10.1016/j.devcel.2009.06.016 19619488 PMC2861485

[B119] Martínez-Estrada O. M. Lettice L. A. Essafi A. Guadix J. A. Slight J. Velecela V. (2010). Wt1 is required for cardiovascular progenitor cell formation through transcriptional control of Snail and E-cadherin. Nat. Genet. 42, 89–93. 10.1038/ng.494 20023660 PMC2799392

[B120] Matsuoka T. Yashiro M. (2018). Biomarkers of gastric cancer: Current topics and future perspective. World J. Gastroenterol. 24, 2818–2832. 10.3748/wjg.v24.i26.2818 30018477 PMC6048430

[B121] Mei J. Liu G. Li R. Xiao P. Yang D. Bai H. (2021). LncRNA SNHG6 knockdown inhibits cisplatin resistance and progression of gastric cancer through miR-1297/BCL-2 axis. Biosci. Rep. 41, BSR20211885. 10.1042/BSR20211885 34821362 PMC8661508

[B122] Meng Q. Wang X. Xue T. Zhao Q. Wang W. Zhao K. (2020). Long noncoding RNA MIR99AHG promotes gastric cancer progression by inducing EMT and inhibiting apoptosis via miR577/FOXP1 axis. Cancer Cell Int. 20, 414. 10.1186/s12935-020-01510-6 32874129 PMC7457246

[B123] Meng Z. Moroishi T. Genes K.-L. G. J. Development (2016). Mechanisms of Hippo pathway regulation. Genes Dev. 30, 1–17. 10.1101/gad.274027.115 26728553 PMC4701972

[B124] Morgan E. Arnold M. Camargo M. C. Gini A. Kunzmann A. T. Matsuda T. (2022). The current and future incidence and mortality of gastric cancer in 185 countries, 2020–40: A population-based modelling study. eClinicalMedicine 47, 101404. 10.1016/j.eclinm.2022.101404, 35497064 PMC9046108

[B125] Mulero M. C. Shahabi S. Ko M. S. Schiffer J. M. Huang D.-B. Wang V. Y.-F. (2018). Protein cofactors are essential for high-affinity DNA binding by the nuclear factor κB RelA subunit. Biochemistry 57, 2943–2957. 10.1021/acs.biochem.8b00158 29708732 PMC5993198

[B126] Necula L. Matei L. Dragu D. Neagu A. I. Mambet C. Nedeianu S. (2019). Recent advances in gastric cancer early diagnosis. World J. Gastroenterol. 25, 2029–2044. 10.3748/wjg.v25.i17.2029 31114131 PMC6506585

[B127] Nie M.-L. Han J. Huang H.-C. Guo T. Huangfu L.-T. Cheng X.-J. (2019). The novel lncRNA p4516 acts as a prognostic biomarker promoting gastric cancer cell proliferation and metastasis. Cancer Manag. Res. 11, 5375–5391. 10.2147/CMAR.S201793 31354346 PMC6578592

[B128] Oh S. C. Sohn B. H. Cheong J.-H. Kim S.-B. Lee J. E. Park K. C. (2018). Clinical and genomic landscape of gastric cancer with a mesenchymal phenotype. Nat. Commun. 9, 1777. 10.1038/s41467-018-04179-8 29725014 PMC5934392

[B129] Pan L. Liang W. Gu J. Zang X. Huang Z. Shi H. (2018). Long noncoding RNA DANCR is activated by SALL4 and promotes the proliferation and invasion of gastric cancer cells. Oncotarget 9, 1915–1930. 10.18632/oncotarget.23019 29416741 PMC5788609

[B130] Pan T. Yu Z. Jin Z. Wu X. Wu A. Hou J. (2021). Tumor suppressor lnc-CTSLP4 inhibits EMT and metastasis of gastric cancer by attenuating HNRNPAB-dependent Snail transcription. Mol. Ther. Nucleic Acids 23, 1288–1303. 10.1016/j.omtn.2021.02.003 33717650 PMC7907227

[B131] Pastar I. Stojadinovic O. Yin N. C. Ramirez H. Nusbaum A. G. Sawaya A. (2014). Epithelialization in wound healing: A comprehensive review. Adv. Wound Care 3, 445–464. 10.1089/wound.2013.0473 PMC4086220 25032064

[B132] Pavlič A. Hauptman N. Boštjančič E. Zidar N. (2022). Long non-coding RNAs as potential regulators of EMT-related transcription factors in colorectal cancer-A systematic review and bioinformatics analysis. Cancers 14, 2280. 10.3390/cancers14092280 35565409 PMC9105237

[B133] Peng W.-X. Koirala P. Oncogene Y.-Y. M. J. (2017). LncRNA-mediated regulation of cell signaling in cancer. Oncogene 36, 5661–5667. 10.1038/onc.2017.184 28604750 PMC6450570

[B134] Peng Z. Wang C.-X. Fang E.-H. Wang G.-B. Tong Q. (2014). Role of epithelial-mesenchymal transition in gastric cancer initiation and progression. World J. Gastroenterol. 20, 5403–5410. 10.3748/wjg.v20.i18.5403 24833870 PMC4017055

[B135] Petkevicius V. Thon C. Steponaitiene R. Skieceviciene J. Janciauskas D. Jechorek D. (2022). Differential expression of long noncoding RNA HOTAIR in intestinal metaplasia and gastric cancer. Clin. Transl. Gastroenterol. 13, e00483. 10.14309/ctg.0000000000000483 35347094 PMC9132515

[B136] Piao H. Y. Guo S. Jin H. Wang Y. Zhang J. (2021). LINC00184 involved in the regulatory network of ANGPT2 via ceRNA mediated miR-145 inhibition in gastric cancer. J. Cancer 12, 2336–2350. 10.7150/jca.49138 33758610 PMC7974899

[B137] Przygodzka P. Papiewska-Pająk I. Bogusz-Koziarska H. Sochacka E. Boncela J. Kowalska M. A. (2019). Regulation of miRNAs by Snail during epithelial-to-mesenchymal transition in HT29 colon cancer cells. Sci. Rep. 9, 2165. 10.1038/s41598-019-39200-7 30770873 PMC6377707

[B138] Qi F. Liu X. Wu H. Yu X. Wei C. Huang X. (2017). Long noncoding AGAP2-AS1 is activated by SP1 and promotes cell proliferation and invasion in gastric cancer. J. Hematol. Oncol. 10, 48. 10.1186/s13045-017-0420-4 28209205 PMC5314629

[B139] Qi M. Yu B. Yu H. Li F. (2020). Integrated analysis of a ceRNA network reveals potential prognostic lncRNAs in gastric cancer. Cancer Med. 9, 1798–1817. 10.1002/cam4.2760 31923354 PMC7050084

[B140] Rao J. Fu J. Meng C. Huang J. Qin X. Zhuang S. (2021). LncRNA SNHG3 promotes gastric cancer cells proliferation, migration, and invasion by targeting miR-326. J. Oncol. 2021, 9935410. 10.1155/2021/9935410 34257656 PMC8260314

[B141] Rawla P. Barsouk A. (2019). Epidemiology of gastric cancer: Global trends, risk factors and prevention. Prz. Gastroenterol. 14, 26–38. 10.5114/pg.2018.80001 30944675 PMC6444111

[B142] Ren J. Xu N. Zhou R. Huang F. Zhang H. Li W. (2021). Long non-coding RNA PCED1B antisense RNA 1 promotes gastric cancer progression via modulating microRNA-215-3p/C-X-C motif chemokine receptor 1 axis. Bioengineered 12, 6083–6095. 10.1080/21655979.2021.1971503 34516330 PMC8806612

[B143] Riihimäki M. Hemminki A. Sundquist K. Sundquist J. Hemminki K. (2016). Metastatic spread in patients with gastric cancer. Oncotarget 7, 52307–52316. 10.18632/oncotarget.10740 27447571 PMC5239553

[B144] Rui Z. Yanli Z. Xin Z. Yongmei Y. Xin Z. Xiaohui L. (2018). Exosomal long noncoding RNA HOTTIP as potential novel diagnostic and prognostic biomarker test for gastric cancer. Mol. Cancer 17, 68. 10.1186/s12943-018-0817-x 29486794 PMC6389063

[B145] Shuai Y. Ma Z. Liu W. Yu T. Yan C. Jiang H. (2020). TEAD4 modulated LncRNA MNX1-AS1 contributes to gastric cancer progression partly through suppressing BTG2 and activating BCL2. Mol. Cancer 19, 6. 10.1186/s12943-019-1104-1 31924214 PMC6953272

[B146] Soltani R. Amini M. Mazaheri Moghaddam M. Jebelli A. Ahmadiyan S. Bidar N. (2022). LncRNA DLGAP1-AS2 overexpression associates with gastric tumorigenesis: A promising diagnostic and therapeutic target. Mol. Biol. Rep. 49, 6817–6826. 10.1007/s11033-021-07038-w 34981339

[B147] Sona M. F. Myung S. K. Park K. Jargalsaikhan G. (2018). Type 1 diabetes mellitus and risk of cancer: A meta-analysis of observational studies. Jpn. J. Clin. Oncol. 48, 426–433. 10.1093/jjco/hyy047 29635473

[B148] Song Z. Wu Y. Yang J. Yang D. Fang X. (2017). Progress in the treatment of advanced gastric cancer. Tumour Biol. 39, 1010428317714626. 10.1177/1010428317714626 28671042

[B149] Stamos J. L. Weis W. I. (2013). The β-catenin destruction complex. Cold Spring Harb. Perspect. Biol. 5, a007898. 10.1101/cshperspect.a007898 23169527 PMC3579403

[B150] Statello L. Guo C.-J. Chen L.-L. Huarte M. (2021). Gene regulation by long non-coding RNAs and its biological functions. Nat. Rev. Mol. Cell Biol. 22, 96–118. 10.1038/s41580-020-00315-9 33353982 PMC7754182

[B151] Stegmann K. Boecker J. Kosan C. Ermert A. Kunz J. Koch M. C. (1999). Human transcription factor SLUG: Mutation analysis in patients with neural tube defects and identification of a missense mutation (D119E) in the slug subfamily-defining region. Mutat. Res. 406, 63–69. 10.1016/s1383-5726(99)00002-3 10479723

[B152] Stemmler M. P. Eccles R. L. Brabletz S. Brabletz T. (2019). Non-redundant functions of EMT transcription factors. Nat. Cell Biol. 21, 102–112. 10.1038/s41556-018-0196-y 30602760

[B153] Sun J. G. Li X. B. Yin R. H. Li X. F. (2020). lncRNA VIM-AS1 promotes cell proliferation, metastasis and epithelial-mesenchymal transition by activating the Wnt/β-catenin pathway in gastric cancer. Mol. Med. Rep. 22, 4567–4578. 10.3892/mmr.2020.11577 33173977 PMC7646824

[B154] Sun J. Song Y. Chen X. Zhao J. Gao P. Huang X. (2015). Novel long non-coding RNA RP11-119F7.4 as a potential biomarker for the development and progression of gastric cancer. Oncol. Lett. 10, 115–120. 10.3892/ol.2015.3186 26170986 PMC4486926

[B155] Sun Q. Hao Q. Prasanth K. V. (2018). Nuclear long noncoding RNAs: Key regulators of gene expression. Trends Genet. 34, 142–157. 10.1016/j.tig.2017.11.005 29249332 PMC6002860

[B156] Sun Q. Li J. Li F. Li H. Bei S. Zhang X. (2019a). LncRNA LOXL1-AS1 facilitates the tumorigenesis and stemness of gastric carcinoma via regulation of miR-708-5p/USF1 pathway. Cell Prolif. 52, e12687. 10.1111/cpr.12687 31468594 PMC6869681

[B157] Sun Y. Han C. (2020). Long non-coding RNA TMPO-as1 promotes cell migration and invasion by sponging MIR-140-5p and inducing SOX4-mediated EMT in gastric cancer. Cancer Manag. Res. 12, 1261–1268. 10.2147/CMAR.S235898 32110100 PMC7039077

[B158] Sun Y. Zhu Q. Yang W. Shan Y. Yu Z. Zhang Q. (2019b). LncRNA H19/miR-194/PFTK1 axis modulates the cell proliferation and migration of pancreatic cancer. J. Cell. Biochem. 120, 3874–3886. 10.1002/jcb.27669 30474270

[B159] Sung H. Ferlay J. Siegel R. L. Laversanne M. Soerjomataram I. Jemal A. (2021). Global cancer statistics 2020: GLOBOCAN estimates of incidence and mortality worldwide for 36 cancers in 185 countries. Ca. Cancer J. Clin. 71, 209–249. 10.3322/caac.21660 33538338

[B160] Syed V. (2016). TGF-Β signaling in cancer. J. Cell. Biochem. 117, 1279–1287. 10.1002/jcb.25496 26774024

[B161] Takei S. Kawazoe A. Shitara K. (2022). The new era of immunotherapy in gastric cancer. Cancers (Basel) 14, 1054. 10.3390/cancers14041054 35205802 PMC8870470

[B162] Takeichi M. Nimura K. Mori M. Nakagami H. Kaneda Y. (2013). The transcription factors Tbx18 and Wt1 control the epicardial epithelial-mesenchymal transition through bi-directional regulation of Slug in murine primary epicardial cells. PLoS One 8, e57829. 10.1371/journal.pone.0057829 23469079 PMC3585213

[B163] Tang H. Massi D. Hemmings B. A. Mandalà M. Hu Z. Wicki A. (2016). AKT-ions with a TWIST between EMT and MET. Oncotarget 7, 62767–62777. 10.18632/oncotarget.11232 27623213 PMC5308764

[B164] Timmerman L. A. Grego-Bessa J. Raya A. Bertrán E. Pérez-Pomares J. M. Díez J. (2004). Notch promotes epithelial-mesenchymal transition during cardiac development and oncogenic transformation. Genes Dev. 18, 99–115. 10.1101/gad.276304 14701881 PMC314285

[B165] Trelstad R. L. Hay E. D. Revel J. D. (1967). Cell contact during early morphogenesis in the chick embryo. Dev. Biol. 16, 78–106. 10.1016/0012-1606(67)90018-8 6035571

[B166] Uemura N. Okamoto S. Yamamoto S. Matsumura N. Yamaguchi S. Yamakido M. (2001). *Helicobacter pylori* infection and the development of gastric cancer. N. Engl. J. Med. 345, 784–789. 10.1056/NEJMoa001999 11556297

[B167] Villarejo A. Cortés-Cabrera A. Molina-Ortíz P. Portillo F. Cano A. (2014). Differential role of Snail1 and Snail2 zinc fingers in E-cadherin repression and epithelial to mesenchymal transition. J. Biol. Chem. 289, 930–941. 10.1074/jbc.M113.528026 24297167 PMC3887216

[B168] Wang F. Zhu W. Yang R. Xie W. Wang D. (2019a). LncRNA ZEB2-AS1 contributes to the tumorigenesis of gastric cancer via activating the Wnt/β-catenin pathway. Mol. Cell. Biochem. 456, 73–83. 10.1007/s11010-018-03491-7 30635820

[B169] Wang H. Di X. Bi Y. Sun S. Wang T. (2021a). Long non-coding RNA LINC00649 regulates YES-associated protein 1 (YAP1)/Hippo pathway to accelerate gastric cancer (GC) progression via sequestering miR-16-5p. Bioengineered 12, 1791–1802. 10.1080/21655979.2021.1924554 33975517 PMC8806528

[B170] Wang H. Dong X. Gu X. Qin R. Jia H. One J. G. J. P. (2015). The MicroRNA-217 functions as a potential tumor suppressor in gastric cancer by targeting GPC5. PLoS One 10, e0125474. 10.1371/journal.pone.0125474 26098560 PMC4476558

[B171] Wang J. Hu W. Wu X. Wang K. Yu J. Luo B. (2016a). CXCR1 promotes malignant behavior of gastric cancer cells *in vitro* and *in vivo* in AKT and ERK1/2 phosphorylation. Int. J. Oncol. 48, 2184–2196. 10.3892/ijo.2016.3428 26983663

[B172] Wang J. Lv B. Su Y. Wang X. Bu J. Oncotargets L. Y. J. (2019b). THERAPYExosome-mediated transfer of lncRNA HOTTIP promotes cisplatin resistance in gastric cancer cells by regulating HMGA1/miR-218 Axis. Onco. Targets. Ther. 12, 11325–11338. 10.2147/OTT.S231846 31908497 PMC6930390

[B173] Wang J. Wang Q. Wei B. Zhou Y. Qian Z. Gao Y. (2019c). Intronic polymorphisms in genes LRFN2 (rs2494938) and DNAH11 (rs2285947) are prognostic indicators of esophageal squamous cell carcinoma. BMC Med. Genet. 20, 72. 10.1186/s12881-019-0796-9 31053115 PMC6499982

[B174] Wang S. Liu F. Deng J. Cai X. Han J. Liu Q. (2016b). Long noncoding RNA ROR regulates proliferation, invasion, and stemness of gastric cancer stem cell. Cell. Reprogr. 18, 319–326. 10.1089/cell.2016.0001 27602437

[B175] Wang X. Gong Z. Ma L. Wang Q. (2021b). LncRNA GACAT1 induces tongue squamous cell carcinoma migration and proliferation via miR-149. J. Cell. Mol. Med. 25, 8215–8221. 10.1111/jcmm.16690 34378327 PMC8419168

[B176] Wang Y. Gao W. J. (2021). Long non-coding RNA-H19 promotes ovarian cancer cell proliferation and migration via the microRNA-140/Wnt1 axis. Kaohsiung J. Med. Sci. 37, 768–775. 10.1002/kjm2.12393 34002485 PMC11896232

[B177] Wang Y. J. Liu J. Z. Lv P. Dang Y. Gao J. Y. Wang Y. (2016c). Long non-coding RNA CCAT2 promotes gastric cancer proliferation and invasion by regulating the E-cadherin and LATS2. Am. J. Cancer Res. 6, 2651–2660. 27904778 PMC5126280

[B178] Wang Y. Shi J. Chai K. Ying X. Zhou B. P. (2013). The role of snail in EMT and tumorigenesis. Curr. Cancer Drug Targets 13, 963–972. 10.2174/15680096113136660102 24168186 PMC4004763

[B179] Wang Y. Zhou B. P. (2013). Epithelial-mesenchymal transition-A hallmark of breast cancer metastasis. Cancer Hallm. 1, 38–49. 10.1166/ch.2013.1004 24611128 PMC3944831

[B180] Wei L. Sun J. Zhang N. Zheng Y. Wang X. Lv L. (2020). Noncoding RNAs in gastric cancer: Implications for drug resistance. Mol. Cancer 19, 62. 10.1186/s12943-020-01185-7 32192494 PMC7081551

[B181] Wei Z. Yajuan Z. Wenbin W. Min C. Chunjie M. (2018). Enhanced expression of lncRNA TP73-AS1 predicts unfavorable prognosis for gastric cancer and promotes cell migration and invasion by induction of EMT. Gene 678, 377–383. 10.1016/j.gene.2018.08.055 30118890

[B182] Wu H. Hu Y. Liu X. Song W. Gong P. Zhang K. (2017). LncRNA TRERNA1 function as an enhancer of SNAI1 promotes gastric cancer metastasis by regulating epithelial-mesenchymal transition. Mol. Ther. Nucleic Acids 8, 291–299. 10.1016/j.omtn.2017.06.021 28918030 PMC5537167

[B183] Wu P. Mo Y. Peng M. Tang T. Zhong Y. Deng X. (2020). Emerging role of tumor-related functional peptides encoded by lncRNA and circRNA. Mol. Cancer 19, 22. 10.1186/s12943-020-1147-3 32019587 PMC6998289

[B184] Wu Q. Xiang S. Ma J. Hui P. Wang T. Meng W. (2018). Long non-coding RNA CASC15 regulates gastric cancer cell proliferation, migration and epithelial mesenchymal transition by targeting CDKN1A and ZEB1. Mol. Oncol. 12, 799–813. 10.1002/1878-0261.12187 29489064 PMC5983148

[B185] Xie M. Nie F. Q. Sun M. Xia R. Liu Y. W. Zhou P. (2015). Decreased long noncoding RNA SPRY4-IT1 contributing to gastric cancer cell metastasis partly via affecting epithelial-mesenchymal transition. J. Transl. Med. 13, 250. 10.1186/s12967-015-0595-9 26238992 PMC4522960

[B186] Xiu B. Chi Y. Liu L. Chi W. Zhang Q. Chen J. (2019). LINC02273 drives breast cancer metastasis by epigenetically increasing AGR2 transcription. Mol. Cancer 18, 187. 10.1186/s12943-019-1115-y 31856843 PMC6921600

[B187] Xu G. Meng L. Yuan D. Li K. Zhang Y. Dang C. (2018a). MEG3/miR-21 axis affects cell mobility by suppressing epithelial-mesenchymal transition in gastric cancer. Oncol. Rep. 40, 39–48. 10.3892/or.2018.6424 29749532 PMC6059753

[B188] Xu M. D. Wang Y. Weng W. Wei P. Qi P. Zhang Q. (2017). A positive feedback loop of lncRNA-PVT1 and FOXM1 facilitates gastric cancer growth and invasion. Clin. Cancer Res. 23, 2071–2080. 10.1158/1078-0432.CCR-16-0742 27756785

[B189] Xu Q. Deng F. Qin Y. Zhao Z. Wu Z. Xing Z. (2016). Long non-coding RNA regulation of epithelial–mesenchymal transition in cancer metastasis. Cell Death Dis. 7, e2254. 10.1038/cddis.2016.149 27277676 PMC5143379

[B190] Xu W. He L. Li Y. Tan Y. Zhang F. Hong Xu %J Bioscience B. (2018b). Silencing of lncRNA ZFAS1 inhibits malignancies by blocking Wnt/β-catenin signaling in gastric cancer cells. Biosci. Biotechnol. Biochem. 82, 456–465. 10.1080/09168451.2018.1431518 29424266

[B191] Xu W. Yang Z. Adhesion N. L. J. C. Migration (2015). A new role for the PI3K/Akt signaling pathway in the epithelial-mesenchymal transition. Cell adh. Migr. 9, 317–324. 10.1080/19336918.2015.1016686 26241004 PMC4594353

[B192] Xu Y. Sun J. Y. Jin Y. F. Yu H. (2018c). PCAT6 participates in the development of gastric cancer through endogenously competition with microRNA-30. Eur. Rev. Med. Pharmacol. Sci. 22, 5206–5213. 10.26355/eurrev_201808_15718 30178843

[B193] Xu Y. Zhang G. Zou C. Qi W. Gong Z. Zhang G. (2019). Long non-coding RNA LINC01225 promotes proliferation, invasion and migration of gastric cancer via Wnt/β-catenin signalling pathway. J. Cell. Mol. Med. 23, 7581–7591. 10.1111/jcmm.14627 31460694 PMC6815774

[B194] Xu Z. Y. Yu Q. M. Du Y. A. Yang L. T. Dong R. Z. Huang L. (2013). Knockdown of long non-coding RNA HOTAIR suppresses tumor invasion and reverses epithelial-mesenchymal transition in gastric cancer. Int. J. Biol. Sci. 9, 587–597. 10.7150/ijbs.6339 23847441 PMC3708039

[B195] Yan K. Tian J. Shi W. Xia H. Zhu Y. (2017). LncRNA SNHG6 is associated with poor prognosis of gastric cancer and promotes cell proliferation and EMT through epigenetically silencing p27 and sponging miR-101-3p. Cell. Physiol. biochem. 42, 999–1012. 10.1159/000478682 28683446

[B196] Yang J. Antin P. Berx G. Blanpain C. Brabletz T. Bronner M. (2020a). Guidelines and definitions for research on epithelial–mesenchymal transition. Nat. Rev. Mol. Cell Biol. 21, 341–352. 10.1038/s41580-020-0237-9 32300252 PMC7250738

[B197] Yang L. Cui M. Zhang L. Song L. (2017). FOXM1 facilitates gastric cancer cell migration and invasion by inducing Cathepsin D. Oncotarget 8, 68180–68190. 10.18632/oncotarget.19254 28978107 PMC5620247

[B198] Yang L. Ying X. Liu S. Lyu G. Xu Z. Zhang X. (2020b). Gastric cancer: Epidemiology, risk factors and prevention strategies. Chin. J. Cancer Res. 32, 695–704. 10.21147/j.issn.1000-9604.2020.06.03 33446993 PMC7797232

[B199] Yang T. Zeng H. Chen W. Zheng R. Zhang Y. Li Z. (2016a). *Helicobacter pylori* infection, H19 and LINC00152 expression in serum and risk of gastric cancer in a Chinese population. Cancer Epidemiol. 44, 147–153. 10.1016/j.canep.2016.08.015 27592063

[B200] Yang X.-Z. Cheng T.-T. He Q.-J. Lei Z.-Y. Chi J. Tang Z. (2018). LINC01133 as ceRNA inhibits gastric cancer progression by sponging miR-106a-3p to regulate APC expression and the Wnt/β-catenin pathway. Mol. Cancer 17, 126. 10.1186/s12943-018-0874-1 30134915 PMC6106894

[B201] Yang Y. Shao Y. Zhu M. Li Q. Yang F. Lu X. (2016b). Using gastric juice lncRNA-ABHD11-AS1 as a novel type of biomarker in the screening of gastric cancer. Tumour Biol. 37, 1183–1188. 10.1007/s13277-015-3903-3 26280398

[B202] Ye M. Zhang J. Wei M. Liu B. Dong K. (2020). Emerging role of long noncoding RNA-encoded micropeptides in cancer. Cancer Cell Int. 20, 506. 10.1186/s12935-020-01589-x 33088214 PMC7565808

[B203] Ye Y. Ge O. Zang C. Yu L. Eucker J. Chen Y. (2022). LINC01094 predicts poor prognosis in patients with gastric cancer and is correlated with EMT and macrophage infiltration. Technol. Cancer Res. Treat. 21, 15330338221080977. 10.1177/15330338221080977 35254147 PMC8905065

[B204] Yilmaz M. Christofori G. (2009). EMT, the cytoskeleton, and cancer cell invasion. Cancer Metastasis Rev. 28, 15–33. 10.1007/s10555-008-9169-0 19169796

[B205] Yin G. Tian P. R. He A. B. Yan W. Li T. X. Sun Z. P. (2020). Lncrna linc00689 promotes the progression of gastric cancer through upregulation of adam9 by sponging mir-526b-3p. Cancer Manag. Res. 12, 4227–4239. 10.2147/CMAR.S231042 32581594 PMC7280092

[B206] Ying C. Guoqing W. Hongwei X. Huangfei Y. Qiuling T. Feng B. (2017a). Down regulation of lincRNA-p21 contributes to gastric cancer development through Hippo-independent activation of YAP. Oncotarget 8, 63813–63824. 10.18632/oncotarget.19130 28969031 PMC5609963

[B207] Ying Y. Qingwu L. Mingming X. Zhenju S. Chaoyang T. Physiology T. Z. J. C. (2017b). Emodin: One main ingredient of shufeng jiedu capsule reverses chemoresistance of lung cancer cells through inhibition of EMT. Cell. Physiol. biochem. 42, 1063–1072. 10.1159/000478754 28662514

[B208] Yu L. Chun-Dong Z. Cheng Z. Dong-Qiu D. (2020). DLX6-AS1/miR-204-5p/OCT1 positive feedback loop promotes tumor progression and epithelial-mesenchymal transition in gastric cancer. Gastric Cancer 23, 212–227. 10.1007/s10120-019-01002-1 31463827

[B209] Yu Y. Li L. Zheng Z. Chen S. Chen E. Hu Y. (2017). Long non-coding RNA linc00261 suppresses gastric cancer progression via promoting Slug degradation. J. Cell. Mol. Med. 21, 955–967. 10.1111/jcmm.13035 27878953 PMC5387161

[B210] Yuan H. Kajiyama H. Ito S. Yoshikawa N. Hyodo T. Asano E. (2013). ALX1 induces snail expression to promote epithelial-to-mesenchymal transition and invasion of ovarian cancer cells. Cancer Res. 73, 1581–1590. 10.1158/0008-5472.CAN-12-2377 23288509

[B211] Zeisberg M. Neilson E. G. (2009). Biomarkers for epithelial-mesenchymal transitions. J. Clin. Invest. 119, 1429–1437. 10.1172/JCI36183 19487819 PMC2689132

[B212] Zeng L. Liao Q. Zou Z. Wen Y. Wang J. Liu C. (2018). Long non-coding RNA XLOC_006753 promotes the development of multidrug resistance in gastric cancer cells through the PI3K/AKT/mTOR signaling pathway. Cell. Physiol. biochem. 51, 1221–1236. 10.1159/000495499 30481766

[B213] Zhan T. Rindtorff N. Oncogene M. B. J. (2017). Wnt signaling in cancer. Oncogene 36, 1461–1473. 10.1038/onc.2016.304 27617575 PMC5357762

[B214] Zhang C. Guo F. Xu G. Ma J. Shao F. (2015). STAT3 cooperates with Twist to mediate epithelial-mesenchymal transition in human hepatocellular carcinoma cells. Oncol. Rep. 33, 1872–1882. 10.3892/or.2015.3783 25653024

[B215] Zhang G. Li S. Lu J. Ge Y. Wang Q. Ma G. (2018a). LncRNA MT1JP functions as a ceRNA in regulating FBXW7 through competitively binding to miR-92a-3p in gastric cancer. Mol. Cancer 17, 87. 10.1186/s12943-018-0829-6 29720189 PMC5930724

[B216] Zhang J. Jin H. Y. Wu Y. Zheng Z. C. Guo S. Wang Y. (2019a). Hypoxia-induced LncRNA PCGEM1 promotes invasion and metastasis of gastric cancer through regulating SNAI1. Clin. Transl. Oncol. 21, 1142–1151. 10.1007/s12094-019-02035-9 30690667

[B217] Zhang K. Lu C. Huang X. Cui J. Li J. Gao Y. (2019b). Long noncoding RNA AOC4P regulates tumor cell proliferation and invasion by epithelial-mesenchymal transition in gastric cancer. Ther. Adv. Gastroenterol. 12, 1756284819827697. 10.1177/1756284819827697 PMC6383096 30815034

[B218] Zhang W. Zhan F. Li D. Wang T. Huang H. (2020a). RGMB-AS1/miR-22-3p/NFIB axis contributes to the progression of gastric cancer. Neoplasma 67, 484–491. 10.4149/neo_2020_190418N350 32064882

[B219] Zhang X. Liu G. Ding L. Jiang T. Shao S. Gao Y. (2018b). HOXA3 promotes tumor growth of human colon cancer through activating EGFR/Ras/Raf/MEK/ERK signaling pathway. J. Cell. Biochem. 119, 2864–2874. 10.1002/jcb.26461 29073728

[B220] Zhang X. Wu J. (2021). LINC00665 promotes cell proliferation, invasion, and metastasis by activating the TGF-β pathway in gastric cancer. Pathol. Res. Pract. 224, 153492. 10.1016/j.prp.2021.153492 34091388

[B221] Zhang Y. Yuan Y. Zhang Y. Cheng L. Zhou X. Chen K. (2020b). SNHG7 accelerates cell migration and invasion through regulating miR-34a-Snail-EMT axis in gastric cancer. Cell Cycle 19, 142–152. 10.1080/15384101.2019.1699753 31814518 PMC6927713

[B222] Zhao H. Zhang K. Wang T. Cui J. XI H. Wang Y. (2018). Long non-coding RNA AFAP1-antisense RNA 1 promotes the proliferation, migration and invasion of gastric cancer cells and is associated with poor patient survival. Oncol. Lett. 15, 8620–8626. 10.3892/ol.2018.8389 29805596 PMC5950603

[B223] Zhao Y. Guo Q. Chen J. Hu J. Wang S. Sun Y. (2014). Role of long non-coding RNA HULC in cell proliferation, apoptosis and tumor metastasis of gastric cancer: A clinical and *in vitro* investigation. Oncol. Rep. 31, 358–364. 10.3892/or.2013.2850 24247585

[B224] Zheng H. X. Cai Y. D. Wang Y. D. Cui X. B. Xie T. T. Li W. J. (2013). Fas signaling promotes motility and metastasis through epithelial-mesenchymal transition in gastrointestinal cancer. Oncogene 32, 1183–1192. 10.1038/onc.2012.126 22508480

[B225] Zheng X. Peng B. Wu X. Ye J. Zhao H. Li Y. (2022). Male-specific long non-coding RNA testis-specific transcript, Y-linked 15 promotes gastric cancer cell growth by regulating Wnt family member 1/β-catenin signaling by sponging microRNA let-7a-5p. Bioengineered 13, 8605–8616. 10.1080/21655979.2022.2053814 35287556 PMC9161946

[B226] Zhou C. An N. Cao C. Wang G. (2020a). lncRNA HOXC-AS1 promotes gastric cancer via binding eIF4AIII by activating Wnt/β-catenin signaling. J. Gene Med. 22, e3202. 10.1002/jgm.3202 32307743

[B227] Zhou H. Shen W. Zou H. Lv Q. Shao P. (2020b). Circulating exosomal long non-coding RNA H19 as a potential novel diagnostic and prognostic biomarker for gastric cancer. J. Int. Med. Res. 48, 300060520934297. 10.1177/0300060520934297 32660285 PMC7361491

[B228] Zhou Q. Li H. Jing J. Yuan Y. Sun L. (2020c). Evaluation of C5orf66-AS1 as a potential biomarker for predicting early gastric cancer and its role in gastric carcinogenesis. Onco. Targets. Ther. 13, 2795–2805. 10.2147/OTT.S239965 32308414 PMC7136487

[B229] Zhou X. Chen H. Zhu L. Hao B. Zhang W. Hua J. (2016). *Helicobacter pylori* infection related long noncoding RNA (lncRNA) AF147447 inhibits gastric cancer proliferation and invasion by targeting MUC2 and up-regulating miR-34c. Oncotarget 7, 82770–82782. 10.18632/oncotarget.13165 27835575 PMC5347731

[B230] Zhu S. Wang J. He Y. Meng N. Yan G. R. (2018). Peptides/proteins encoded by non-coding RNA: A novel resource bank for drug targets and biomarkers. Front. Pharmacol. 9, 1295. 10.3389/fphar.2018.01295 30483132 PMC6243196

